# Host triacylglycerols shape the lipidome of intracellular trypanosomes and modulate their growth

**DOI:** 10.1371/journal.ppat.1006800

**Published:** 2017-12-27

**Authors:** Felipe Gazos-Lopes, Jessica L. Martin, Peter C. Dumoulin, Barbara A. Burleigh

**Affiliations:** Department of Immunology and Infectious Diseases, Harvard T.H. Chan School of Public Health, Boston, Massachusetts, United States of America; Johns Hopkins School of Public Health, UNITED STATES

## Abstract

Intracellular infection and multi-organ colonization by the protozoan parasite, *Trypanosoma cruzi*, underlie the complex etiology of human Chagas disease. While *T*. *cruzi* can establish cytosolic residence in a broad range of mammalian cell types, the molecular mechanisms governing this process remain poorly understood. Despite the anticipated capacity for fatty acid synthesis in this parasite, recent observations suggest that intracellular *T*. *cruzi* amastigotes may rely on host fatty acid metabolism to support infection. To investigate this prediction, it was necessary to establish baseline lipidome information for the mammalian-infective stages of *T*. *cruzi* and their mammalian host cells. An unbiased, quantitative mass spectrometric analysis of lipid fractions was performed with the identification of 1079 lipids within 30 classes. From these profiles we deduced that *T*. *cruzi* amastigotes maintain an overall lipid identity that is distinguishable from mammalian host cells. A deeper analysis of the fatty acid moiety distributions within each lipid subclass facilitated the high confidence assignment of host- and parasite-like lipid signatures. This analysis unexpectedly revealed a strong host lipid signature in the parasite lipidome, most notably within its glycerolipid fraction. The near complete overlap of fatty acid moiety distributions observed for host and parasite triacylglycerols suggested that *T*. *cruzi* amastigotes acquired a significant portion of their lipidome from host triacylglycerol pools. Metabolic tracer studies confirmed long-chain fatty acid scavenging by intracellular *T*. *cruzi* amastigotes, a capacity that was significantly diminished in host cells deficient for *de novo* triacylglycerol synthesis via the diacylglycerol acyltransferases (DGAT1/2). Reduced *T*. *cruzi* amastigote proliferation in DGAT1/2-deficient fibroblasts further underscored the importance of parasite coupling to host triacylglycerol pools during the intracellular infection cycle. Thus, our comprehensive lipidomic dataset provides a substantially enhanced view of *T*. *cruzi* infection biology highlighting the interplay between host and parasite lipid metabolism with potential bearing on future therapeutic intervention strategies.

## Author summary

The development of human Chagas disease is associated with persistent intracellular infection with the protozoan parasite, *Trypanosoma cruzi*, which displays tropism for tissues with characteristically high fatty acid flux, such as heart and adipose tissues. Previous studies have highlighted fatty acid metabolism as likely critical to support the growth and survival of this intracellular pathogen, however biochemical data supporting this prediction is currently lacking. Employing an untargeted lipid mass spectrometry approach, we defined the lipidome of intracellular *T*. *cruzi* parasites and their mammalian host cells. Comparative analyses revealed that the fatty acid signatures in the triacylglycerol (TG) pools were highly conserved between parasite and host, suggesting a major route of fatty acid acquisition by this pathogen via host TG. Metabolic tracer studies demonstrated intracellular parasite incorporation of exogenous palmitate into both neutral and phospholipid subclasses that was diminished in host cells deficient for TG synthesis. Moreover, parasites grown in these cells displayed reduced proliferation, demonstrating the importance of parasite coupling to host TG pools during the intracellular infection cycle.

## Introduction

Infection with the protozoan parasite *Trypanosoma cruzi* underlies the development of human Chagas disease, a progressive and debilitating condition characterized by cardiac and gastrointestinal disturbances [1]. With an estimated 8 million people infected [2] and limited treatment options [3], this neglected tropical disease remains a significant health and economic burden in Latin America and an emerging immigrant health problem in non-endemic regions of the world [4]. Mammalian infectious forms of *T*. *cruzi* are transmitted in the feces of insect vectors (family Triatominae), congenitally, via oral infection by the consumption of contaminated foods and liquids, or in the blood and organs of infected donors [5], where they colonize a range of cell and tissue types during the acute stage of infection [6, 7]. Immune control mechanisms are insufficient to eliminate infection [8], leading to chronic infection with parasite persistence in a variety of tissues including cardiac muscle [9–11], gastrointestinal smooth muscle [7, 12, 13], and adipose tissue [7, 14, 15]. While *T*. *cruzi* infection can cause acute myocarditis, parasites often persist asymptomatically for decades before clinical symptoms arise in infected individuals [16]. It is now recognized that tissue infection with *T*. *cruzi* is highly dynamic, even during the more tissue-restrictive chronic phase [7]. As such, understanding the mechanisms that underlie the successful intracellular colonization of diverse host cell types by *T*. *cruzi* is a crucial step to elucidating processes involved in the development and progression of both the acute and chronic stages of Chagas disease.

In the mammalian host, *T*. *cruzi* cycles between two morphologically and biochemically distinct forms: non-dividing, motile trypomastigote forms that circulate in the body and actively penetrate cells to establish intracellular infection [17] and obligate intracellular amastigote (ICA) forms that replicate in the host cell cytosol [18]. Like other intracellular pathogens (e.g., [19]), *T*. *cruzi* amastigotes must meet their metabolic demands by coupling to host metabolic processes. While such metabolic dependencies are potentially exploitable for pathogen control [20, 21], fundamental knowledge of the host pathways that are co-opted by *T*. *cruzi* amastigotes during infection is lacking. To address this gap, we previously performed a genome-wide RNA interference screen in human cells to identify host factors that are permissive for intracellular *T*. *cruzi* growth [22]. Fatty acid (FA) metabolism emerged among the top host cellular pathways associated with an efficient *T*. *cruzi* amastigote growth phenotype in human cells, where an increase in parasite proliferation was observed under conditions expected to favor FA uptake and oxidation by infected host cells [22]. Given that *T*. *cruzi* upregulates expression of FA oxidation and membrane lipid synthesis enzymes as an early adaptation to the host intracellular environment [23–25], and the noted tropism of this parasite for tissues with high rates of FA metabolism [26], intracellular *T*. *cruzi* amastigotes appear to be poised to exploit host FA metabolism to meet their metabolic needs. However, biochemical evidence to support this prediction is currently lacking. In this study, we have leveraged comprehensive lipid mass spectrometry to investigate the potential coupling of parasite and host FA metabolism in a cell culture model of *T*. *cruzi* infection. We provide several lines of evidence to demonstrate that cytosolically-localized *T*. *cruzi* amastigotes co-opt long chain fatty acids (LCFA), predominantly from host triacylglycerol (TG) pools, a process that facilitates the replication of this intracellular pathogen.

## Results

### Comprehensive lipidomic analysis of intracellular *T*. *cruzi* amastigotes and mammalian cells reveals distinct lipidome signatures

**Fig 1:**
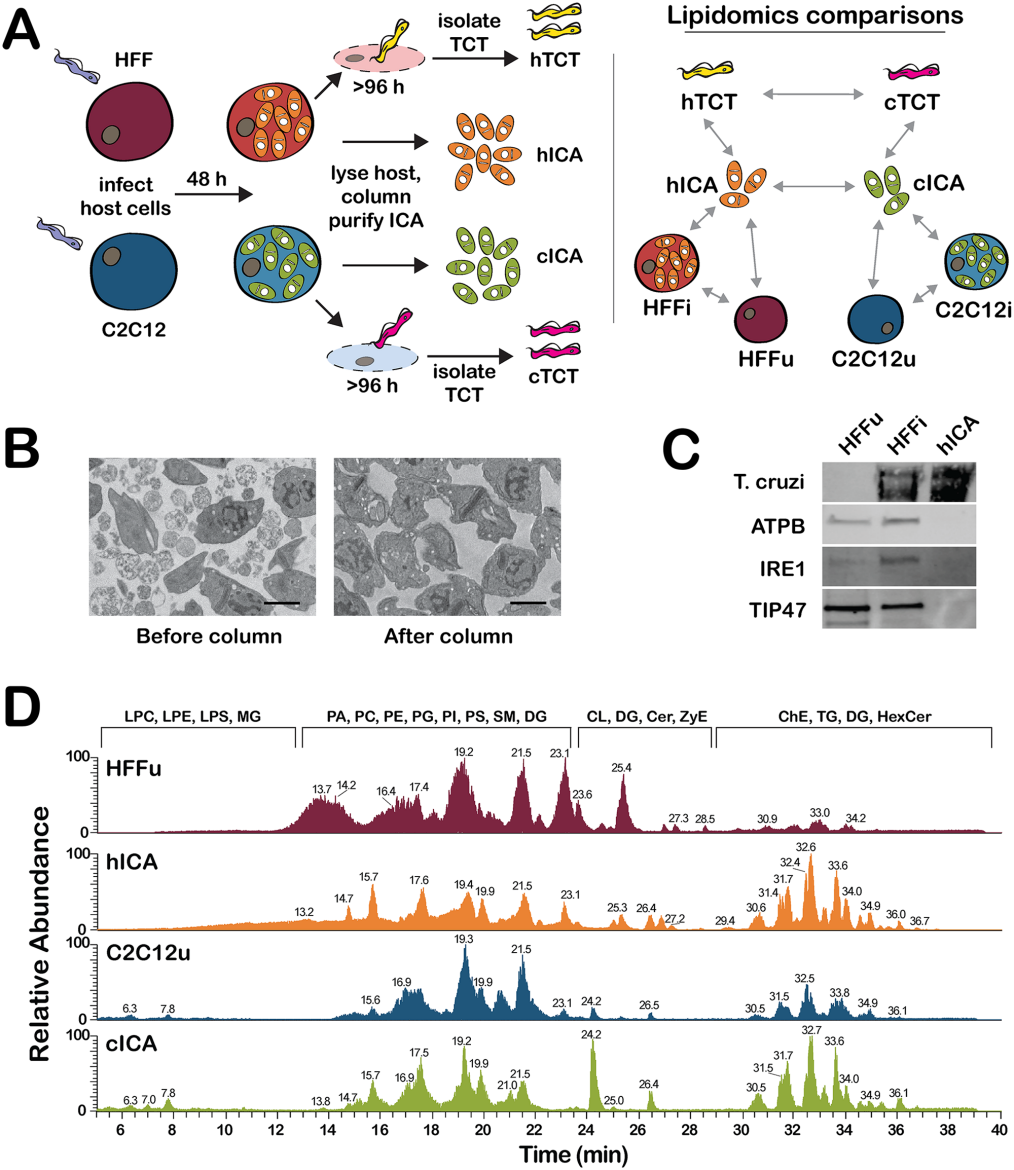
Overview of experimental design, evaluation of *T*. *cruzi* amastigote isolation and LC-MS/MS methods. **(A)** Schematic overview of the experimental approach that involved parallel lipidomics analysis of *T*. *cruzi* parasites isolated from two different mammalian cell types: C2C12 (mouse skeletal myoblast) and HFF (human foreskin fibroblasts). Host cell infection was established with *T*. *cruzi* trypomastigotes (TCT) and intracellular *T*. *cruzi* amastigotes (ICA) were isolated from infected host cells 48 hours post infection as detailed in the Methods. *T*. *cruzi* amastigote purity was evaluated using **(B)** transmission electron microscopy, scale bar = 2 μm, and **(C)** western blot analysis using antibodies to *T*. *cruzi*, host mitochondria (ATP5B), endoplasmic reticulum (IRE1) and lipid droplets (TIP47) to probe whole cell lysates of control uninfected HFF (*HFFu*), *T*. *cruzi*-infected HFF *(HFFi*), and *T*. *cruzi* amastigotes purified from HFF (*hICA*). **(D)** Positive ion mode base peak chromatogram of lipid extracts derived from C2C12, HFF, and cognate *T*. *cruzi* amastigote (cICA and hICA, respectively) analyzed by LC-ESI-MS/MS. The major lipid subclasses eluting at different retention times (min) are indicated above the chromatogram. TG–triacylglycerol, DG–diacylglycerol, Cer–ceramide, CerG–hexosylceramide, SM–sphingomyelin, LPC–lysophosphatidylcholine, PC–phosphatidylcholine, LPE–lysophosphatidylethanolamine, PE–phosphatidylethanolamine, LPS–lysophosphatidylserine, PS–phosphatidylserine, PI–phosphatidylinositol, PG–phosphatidylglycerol.

To assess the potential contribution of host FA metabolism to *T*. *cruzi* amastigote development, it was necessary to first establish steady-state lipidomic signatures for the parasites and for their cognate mammalian host cells. *T*. *cruzi* is capable of infecting and completing its intracellular life cycle in most nucleated mammalian cell types *in vitro* [27]. Anticipating the potential influence of the immediate host cellular environment on the *T*. *cruzi* amastigote lipidome, parallel lipidomic analyses were performed in two different mammalian cell types that have predicted differences in lipid metabolism [28]: human foreskin fibroblasts (HFF) and mouse skeletal myoblasts (C2C12), as outlined (Fig 1A). *T*. *cruzi* amastigotes were isolated from infected cell monolayers at 48 hours post infection (hpi), a time point chosen to reflect a period of active intracellular parasite replication [22]. Isolated parasites were shown to be free of contaminating host cell organelles and membranous material as assessed by transmission electron microscopy (Fig 1B) and western blot analysis (Fig 1C).

Lipid extracts obtained from mock- and parasite-infected HFF and C2C12, *T*. *cruzi* amastigotes liberated from each host cell type, as well as tissue-culture trypomastigotes (TCT) that egress upon completion of the intracellular infection cycle (Fig 1A) were subjected to an ultra-high performance reverse-phase liquid chromatography (UHP-RPLC) system coupled with a Q-Exactive Hybrid Quadrupole-Orbitrap mass spectrometer operating in high-resolution data-dependent full scan MS/MS in negative and positive ion modes. Representative base peak chromatograms generated from total lipid extracts of uninfected C2C12, HFF, and *T*. *cruzi* amastigotes isolated from these cell types revealed clear peak separation and distinct ion profiles (Fig 1D; S1 Fig). The chromatographic profiles for *T*. *cruzi* amastigotes isolated from different mammalian host cell types exhibited strong similarity, particularly in the peak clusters eluting in the 30–36 min range corresponding to triacylglycerols (TG), diacylglycerols (DG), sphingolipids, and cardiolipins (CL), but differ from their respective host cell of origin (Fig 1D). A total of 1079 lipid molecular species were confidently identified after manual curation of LipidSearch-assigned spectra, with coverage of 30 different lipid subclasses, across all samples (S1 and S2 Tables; Supporting Information: Supplemental Methods). Over half of the lipids identified belong to the glycerophospholipid lipid (GP) category (675 unique molecular species), with most of this diversity associated with diacyl glycerophosphocholine (PC) or diacyl- and ether-linked glycerophosphatidylethanolamine (PE) subclasses (236 and 110 unique species, respectively). The glycerolipid (GL) category comprised approximately one-third of the total lipid diversity, with 326 TG, 58 DG, and 6 monoglycerol (MG) species. Sphingolipids, with 58 sphingomyelin (SM) and 43 ceramide (Cer) species comprised ~10% of the total lipids identified (S1 Table).

**Fig 2:**
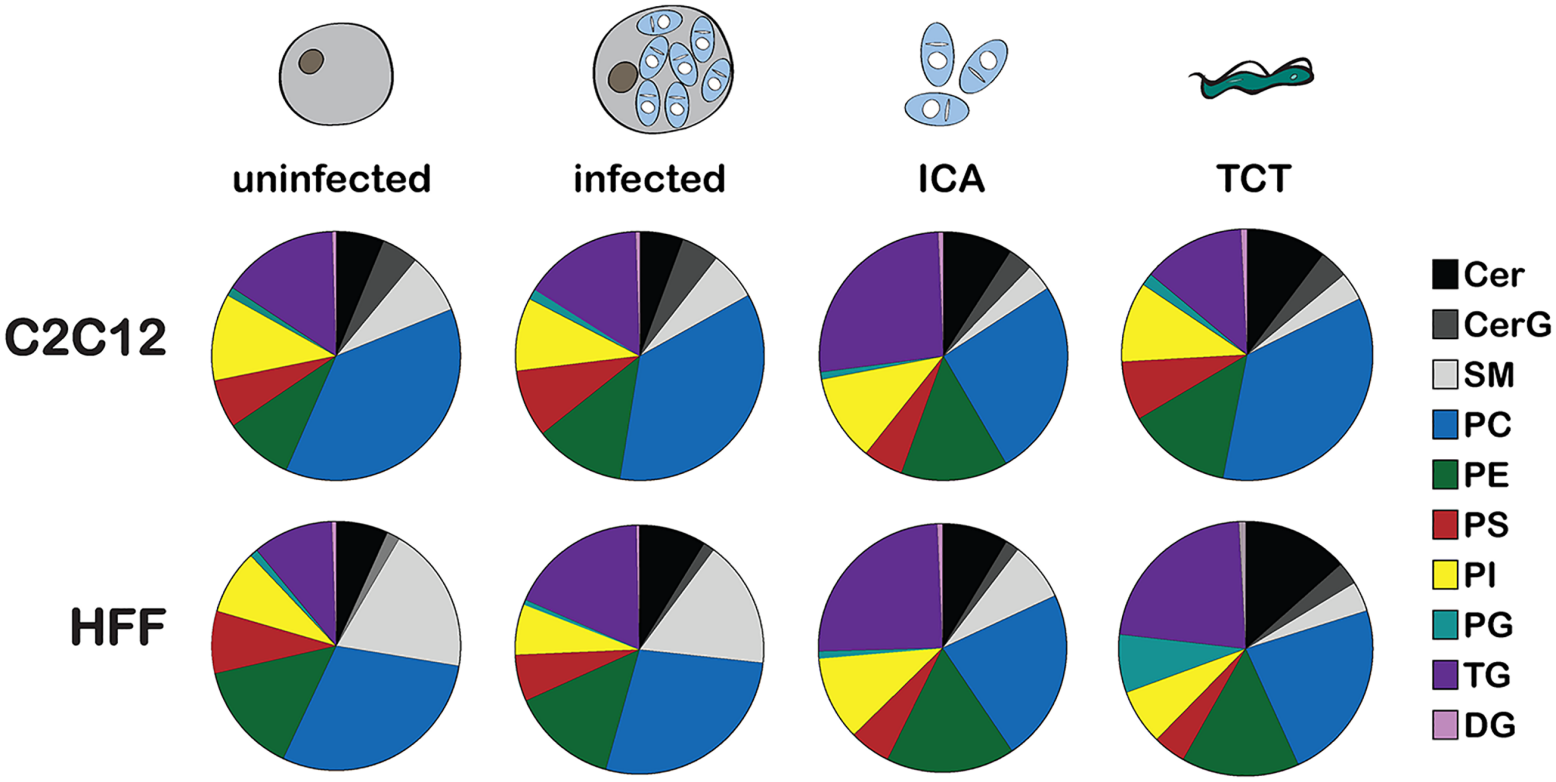
Lipid class breakdown in *T*. *cruzi* and mammalian cells. Pie charts display the relative abundance of the major lipid subclasses of mammalian host cells (C2C12 and HFF) and *T*. *cruzi* intracellular amastigotes (ICA) and tissue-culture trypomastigotes (TCT) represented as a portion of total lipid content in each sample, averaged for 4 independent experiments. TG–triacylglycerol, DG–diacylglycerol, Cer–ceramide, CerG–hexosylceramide, SM–sphingomyelin, PC–phosphatidylcholine, PE–phosphatidylethanolamine, PS–phosphatidylserine, PI–phosphatidylinositol, PG–phosphatidylglycerol.

Next, the proportion of each of the major lipid classes within the lipidomes of *T*. *cruzi* and mammalian cells was plotted to determine if the parasite lipidome could be readily distinguished from that of its host and, if so, what major trends in its lipidome would be conserved regardless of the host cell background (Fig 2). To this end, the relative abundance of each major lipid class identified in *T*. *cruzi* and mammalian cells was compared across all cell types and conditions (Fig 2; S3 Table). As expected, the lipidomes of HFF and C2C12 differ markedly, as evidenced by the quantitative differences in the PC and SM content in these disparate cell types (Fig 2, *uninfected*). Apart from the increased TG content in infected fibroblasts as compared to the uninfected cell (Fig 2, *HFF*), the lipid class distribution of mammalian host cells remained largely unchanged after 48 hours of *T*. *cruzi* infection (Fig 2, *infected*). The lipid class relative abundance profiles of isolated *T*. *cruzi* amastigotes are clearly distinguishable from either mammalian host cell type but display nearly identical profiles independent of host cell of origin (Fig 2, *ICA*). This finding suggests that, at least for the phase of the intracellular *T*. *cruzi* infection cycle examined here, maintenance of a particular lipid class balance may be critical for the growth and survival of this intracellular pathogen. Notably, this lipid identity includes a relatively enriched TG pool in *T*. *cruzi* amastigotes (~25% of total lipid area), reflecting the capacity for lipid storage in this parasite life cycle stage [29]. The lipidomic signatures of the extracellular trypomastigotes diverge from their amastigote counterparts suggestive of developmental regulation of lipid class ratios in this parasite [30]. In addition, the lipidome profiles of *T*. *cruzi* trypomastigotes isolated from distinct mammalian host cell types exhibited marked differences, a feature that may impact future parasite-host interactions. These observations indicate that while the *T*. *cruzi* lipidome is dynamic over the course of its life cycle, the lipid composition requirements may be constrained in the intracellular amastigote stage.

### Principle component analysis reveals complex relationships between host and parasite lipid classes

**Fig 3:**
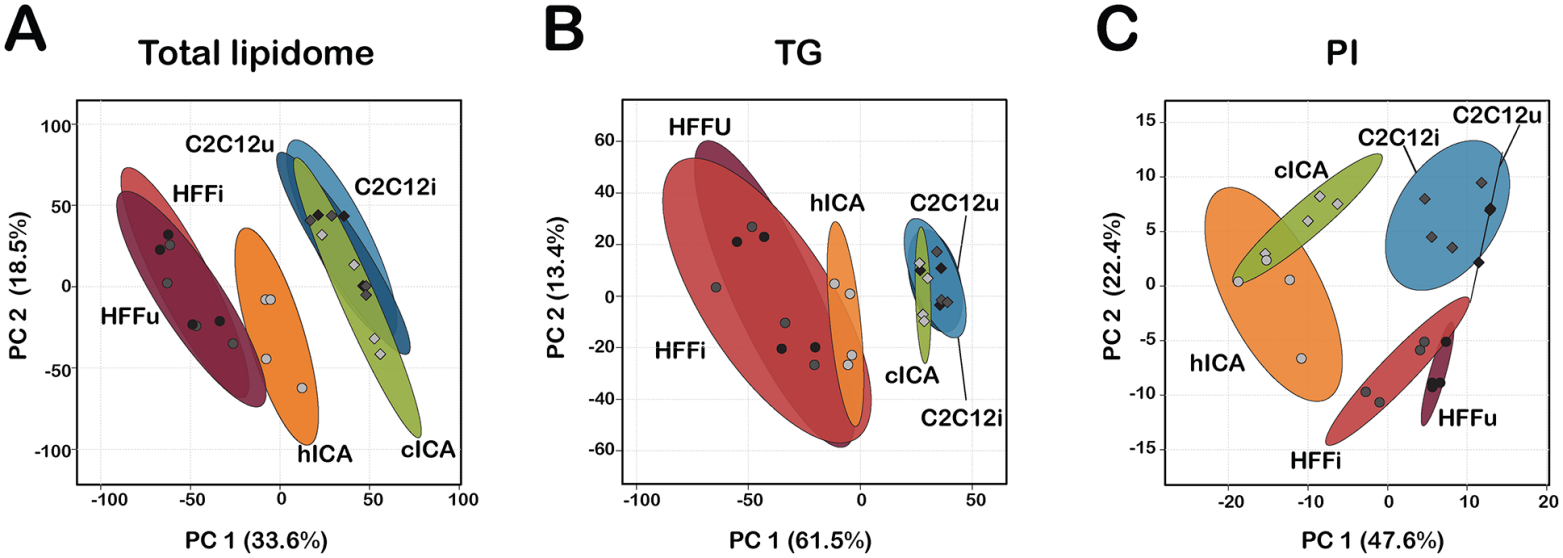
Principle component analysis of host and parasite lipidomes at the lipid species level. Principle component analysis of lipid species are plotted for the **(A)** total lipidome, **(B)** TG subclass, and **(C)** PI subclass. The first two principle components are plotted (PC1 and PC2) with proportion of variance for each component shown in parenthesis. Each sample is represented and the 95% confidence interval indicated in shaded circle.

To compare parasite and host lipidomic signatures in more depth, analyses of the detailed lipid species distributions within each of the major lipid subclasses described above were undertaken. Two-dimensional principal component analyses (PCA) were performed to identify overall trends in the data (Fig 3). When total lipidomes were compared across samples, *T*. *cruzi* amastigotes clustered with their cognate mammalian host cells (Fig 3A) rather than together, as predicted by the proportional lipid class trends observed for *T*. *cruzi* amastigotes isolated from different cell types (Fig 2). To determine if this relationship might be driven by a sub-compartment of the total lipidome, additional PCA plots were generated for individual lipid subclasses. A more complex relationship between host and parasite lipid signatures emerged in these analyses (Fig 3B and 3C; S2 Fig). Within the TG class, tight clustering between *T*. *cruzi* amastigote and cognate mammalian host cell was evident (Fig 3B) whereas, clear divergence between parasite and host lipidomes was observed within the PI pool (Fig 3C). Still other lipid classes showed no distinct trend (S2 Fig). Combined, these data suggest that the steady state lipidome of *T*. *cruzi* amastigotes is a mixture of parasite-synthesized lipids and host-derived lipids obtained through scavenging.

### Intracellular *T*. *cruzi* amastigotes display a mosaic lipidome with signatures of lipid scavenging

*T*. *cruzi* is capable of the synthesis, elongation [31], and desaturation [32] of FA, although the extent to which the intracellular amastigote stage relies on these FA synthesis capacities has not been determined. With several enzymatic activities absent in mammals, *T*. *cruzi* produce some fatty acids that are typically found in much lower abundance in mammalian host cells [32–34]. An example is linoleic acid (C18:2 *cis*,*cis-*Δ^9,12^) which, in a gas chromatography with flame ionization detection (GC-FID) analysis of the esterified FA content of total lipid pools is found to be highly enriched in isolated *T*. *cruzi* amastigotes as compared to mammalian cells (S3 Fig), consistent with previous reports [32, 33]. Conversely, trypanosomes have been reported to have lower levels of oleic acid and palmitic acid than human cells [33, 35]. We therefore predict that lipids produced endogenously by *T*. *cruzi*, either through *de novo* synthesis, or via FA remodeling, will be likely enriched in FA species that are abundant in the parasite (e.g., C18:2; S3 Fig), whereas lipids scavenged from the host and incorporated directly into the parasite lipidome would more closely mirror the FA composition of the mammalian host cell.

**Fig 4:**
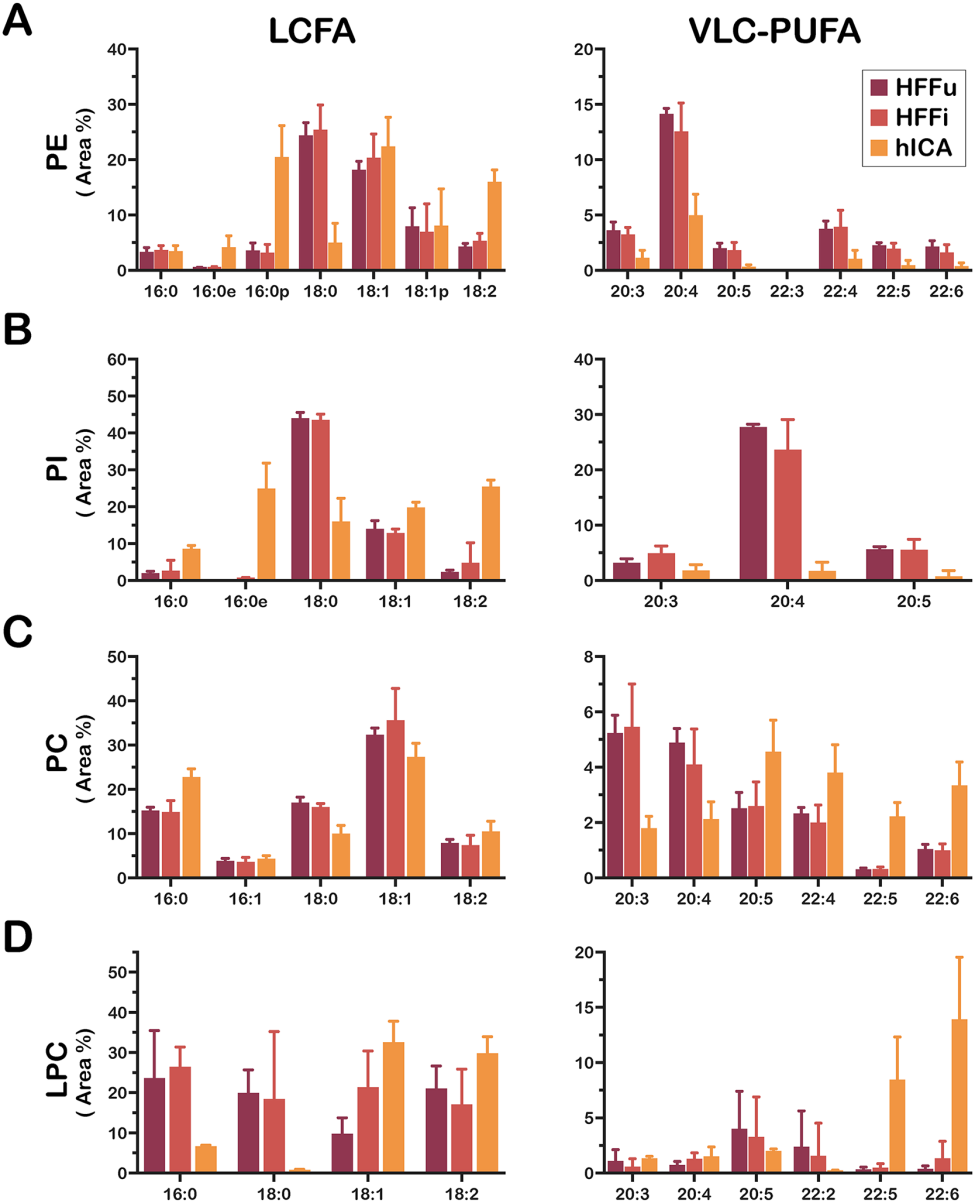
Trends in host and parasite FA composition varies between lipid classes. The relative proportion of identified FA for **(A)** PE, **(B)** PI, **(C)** PC, and **(D)** LPC lipid subclasses is plotted (FA area %; calculated as detailed in Methods) for HFF-derived samples (C2C12-derived samples plotted in S4 Fig): uninfected HFF (*HFFu*), *T*. *cruzi*-infected HFF *(HFFi*) and *T*. *cruzi* amastigotes purified from HFF (*hICA*). The long-chain fatty acid (LCFA) and very long-chain polyunsaturated fatty acid (VLC-PUFA) are plotted separately for clarity. Data are represented as mean ± standard deviation.

With these criteria in mind, a deeper analysis of the parasite and host lipidome data was conducted in which detailed comparisons of the FA moiety composition of each lipid subclass of *T*. *cruzi* and cognate host cells were performed to determine which, if any, parasite lipid classes display signatures indicative of scavenging by *T*. *cruzi* amastigotes. Because of the differences between parasite and mammalian cell FA elongase and desaturase enzymatic machineries mentioned above, emphasis was given to the comparison of long-chain and very long-chain polyunsaturated FA (LC-PUFA and VLC-PUFA, respectively) moiety distributions of each of the major lipid subclasses of *T*. *cruzi* and host. In most cases, this approach identified *T*. *cruzi*-specific trends in the FA moiety distribution of the major GP pools, which were clearly different from the host cells (Fig 4; S4 Fig). For instance, *T*. *cruzi*-derived PE and PI pools had high levels of C18:2 FA, and very low levels of C20:3, C20:4, and C20:5 FA as compared to the host (Fig 4A and 4B). Also, the parasite-derived PE and PI pools exhibited much higher abundance of ether-bound moieties (1-*0-*alkyl and 1*-0-*alkenyl, represented as 16:0e and 16:0p, respectively, in Fig 4A and 4B) as compared to the host. Another parasite-specific trend pertained to the PC and LPC pools, which had markedly higher levels of VLC-PUFA (C22:4, C22:5, and C22:6 FA moieties), than the host (Fig 4C and 4D). Taken together, these data suggest that *T*. *cruzi* amastigotes either do not readily incorporate GP from host cells, preferring to synthesize these lipids *de novo* or extensively remodel FA moieties after acquisition from host GP pools.

**Fig 5:**
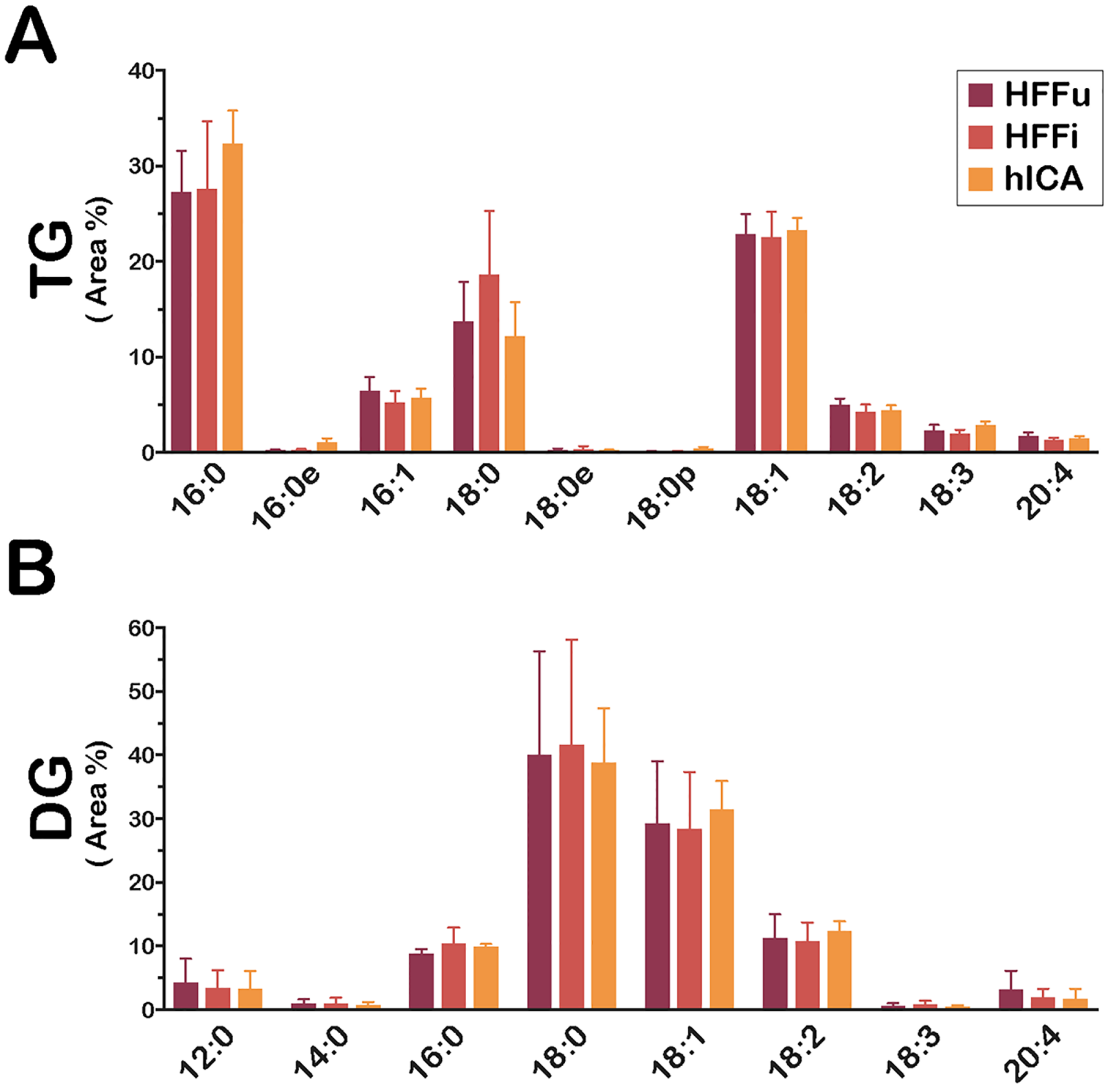
FA composition in TG and DG of *T*. *cruzi* intracellular amastigotes mirrors host cells. FA area % is plotted for **(A)** TG and **(B)** DG classes for HFF-derived samples, (C2C12 plotted in S5 Fig): uninfected HFF (*HFFu*), *T*. *cruzi*-infected HFF *(HFFi*) and *T*. *cruzi* amastigotes purified from HFF (*hICA*). Data are represented as mean ± standard deviation.

In contrast to these observations, the glycerolipid (GL) pools of *T*. *cruzi* amastigotes isolated from HFF (Fig 5) or from C2C12 (S5 Fig) were more similar to their cognate host cell than to each other, in agreement with the PCA plot of the TG subclass (Fig 3B). These trends were made more evident given the divergence between the HFF and C2C12 host cell GL FA composition. This led us to hypothesize that *T*. *cruzi* amastigotes co-opt GL (TG and/or DG) or FA derived from the GL pool, directly from their mammalian host cells.

### *T*. *cruzi* amastigotes acquire and incorporate exogenous FA provided to infected host cells

**Fig 6:**
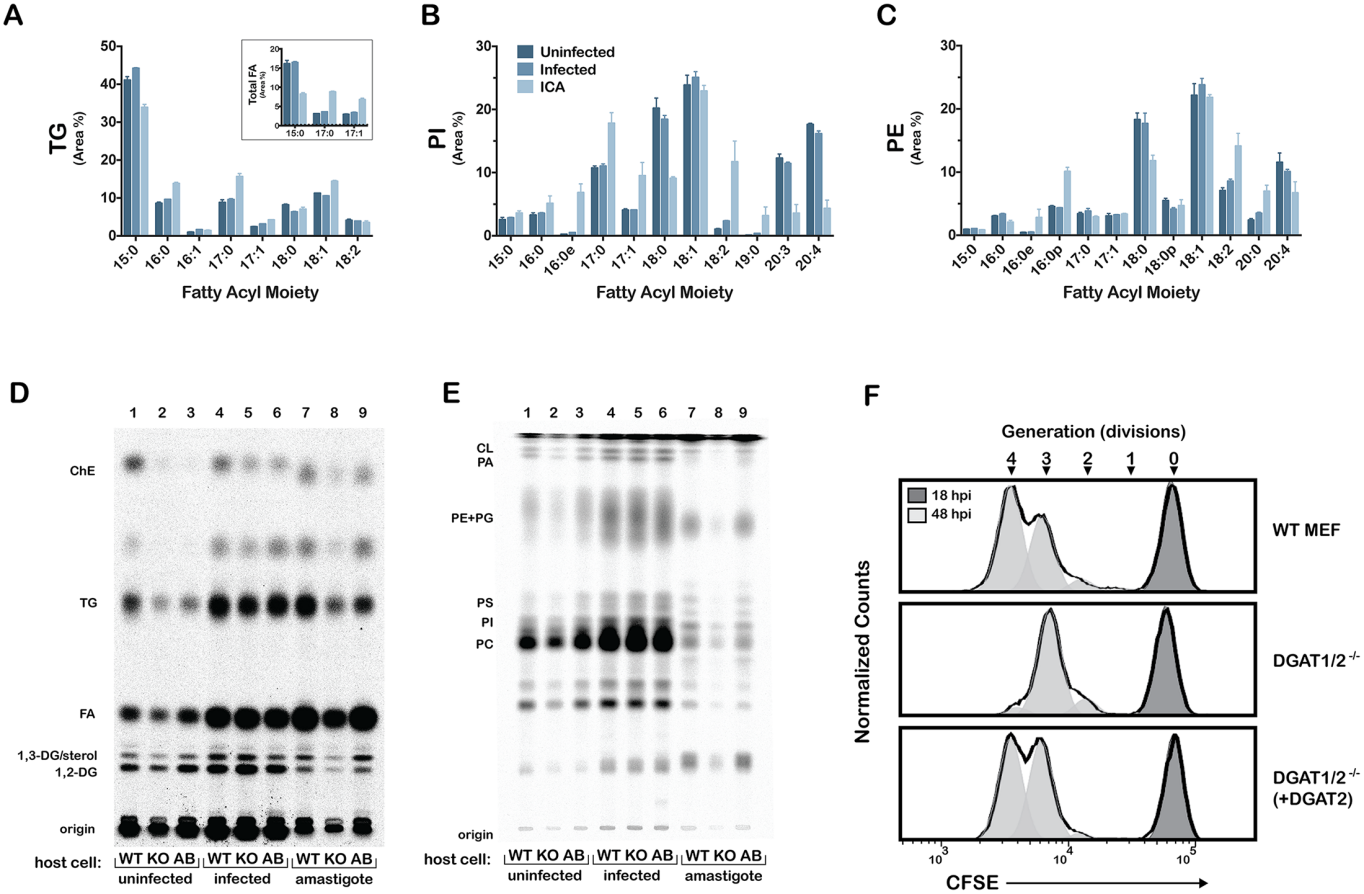
*T*. *cruzi* intracellular amastigotes (ICA) scavenge and incorporate exogenous FA, amastigote FA acquisition and proliferation are compromised in DGAT-TG synthesis deficient host cells. Lipidomic analysis of uninfected HFF (*uninfected*), *T*. *cruzi*-infected HFF *(infected*) and *T*. *cruzi* amastigotes purified from HFF (*ICA*) show incorporation of exogenous C15:0 FA into **(A)** TG, **(A, inset)** total FA, dotted line indicates the average C15:0, C17:0, C17:1 in unlabeled samples, **(B)** PI, and **(C)** PE. Representative autoradiographs showing **(D)** neutral lipid and **(E)** glycerophospholipid TLC analysis of ^14^C-palmitate incorporation into uninfected (lanes 1–3), infected (lanes 4–6), and isolated amastigotes (lanes 7–9) from wild type mouse embryonic fibroblasts, diacylglycerol acyl transferase 1/2 knockout MEF DGAT1/2^-/-^, and DGAT1/2^-/-^ (+DGAT2) cell lines, respectively; **(F)** Proliferation of *T*. *cruzi* amastigotes measured by CFSE intensity at 18 hpi (undivided) and 48 hpi in WT MEF, DGAT1/2^-/-^ and DGAT1/2^-/-^ (+DGAT2) cell lines.

To obtain direct biochemical evidence of FA scavenging by *T*. *cruzi* ICA and investigate whether amastigotes access long chain fatty acid (LCFA) species from their host cells, we used the odd chain fatty acid (OCFA) pentadecanoic acid (C15:0) to trace FA incorporation into different lipid pools of the amastigote. This approach took advantage of the fact that OCFA occur naturally in very low abundance in both *T*. *cruzi* and mammalian cells (~0.3%; of total LCFA abundance, S4 Table). Using our LC-ESI-MS/MS pipeline, we determined the relative abundance of OCFA substituents in the lipidomes of *T*. *cruzi* amastigotes and their host cells after provision of 200 μM C15:0 to *T*. *cruzi*-infected cultures for 6 h (42–48 hpi). This protocol resulted in a striking 30-fold increase in the C15:0 content in total acylated FA in isolated parasites and host cells (Fig 6A, inset), confirming the capacity for C15:0 uptake by mammalian cells, as anticipated [36]. C15:0 was determined to be the most abundant OCFA in the TG subclass (Fig 6A), and was represented in similar levels in host and parasite TG pools, in agreement with the observations from our steady-state lipidomic analyses (Fig 5B).

While acylated C15:0 was found in similar abundance (~15%) in uninfected and infected host cells, isolated intracellular amastigotes showed less C15:0 incorporation (~8%; Fig 6A, inset), Despite the lower C15:0 incorporation in total amastigote FA pool, we observed ~2-fold higher levels of C17:0 and C17:1 in amastigotes than infected or uninfected host cells. This suggests that following import of LCFA from mammalian host cells, *T*. *cruzi* amastigotes are able to modify these fatty acids through the action of parasite elongase and desaturase enzymes [33]. We find that OCFA were unequally distributed across lipid classes in the host and parasite lipidomes, with C15:0 being predominantly incorporated into TG pools in *T*. *cruzi* amastigotes (Fig 6A). In contrast, *T*. *cruzi* amastigote PI was enriched in C17:0 and C17:1, (Fig 6B), however, this enrichment was not observed in amastigote PE, suggesting that a portion of the acquired C15:0 FA was modified by *T*. *cruzi* amastigotes prior to incorporation into different parasite lipid classes. Thus, in conjunction with lipidomic profiling data, metabolic labeling studies provide further evidence that *T*. *cruzi* amastigotes acquire LCFA from host lipid pools and incorporate these FA into their own lipid/membrane synthesis pathways. Furthermore, our results implicate host TG metabolism as a critical factor influencing FA incorporation by *T*. *cruzi* amastigotes.

### Exogenous LCFA flux into intracellular *T*. *cruzi* amastigote lipid pools utilizes host DGAT 1/2-dependent TG synthesis

To investigate the role of host GL metabolism in LCFA scavenging by *T*. *cruzi* amastigotes, mouse embryonic fibroblasts (MEF) deficient in diacylglycerol acyltransferase (DGAT1/2)-dependent TG synthesis were exploited for parasite infection and metabolic labeling studies. *T*. *cruzi*-infected WT, DGAT1/2^-/-^, and DGAT2-complemented DGAT1/2^-/-^ fibroblasts were pulsed with [^14^C(U)]-palmitate for 6 h (42–48 hpi), amastigotes were isolated, and label incorporation into host cell and *T*. *cruzi* amastigote neutral lipids and phospholipids was visualized following separation by TLC (Fig 6D and 6E). As expected from prior characterization of the DGAT1/2^-/-^ fibroblasts [37], the enzyme deficiency resulted in greatly reduced incorporation of exogenous ^14^C-palmitate into TG pools in DGAT1/2^-/-^ cells as compared to WT cells (Fig 6D, lane 1, 2), and was restored with the ectopic expression of DGAT2 (Fig 6D, lane 3). Decreased ^14^C-labeling of cholesterol esters (ChE) was also noted in the DGAT1/2^-/-^ fibroblasts relative to WT cells, a likely consequence of impaired lipid droplet formation in the knockout cells [37]. Interestingly, we observed a marked increase in TG ^14^C-labeling in DGAT1/2^-/-^ cells upon infection with *T*. *cruzi*, suggesting that a non-DGAT TG synthesis pathway (as discussed in [37]) may be upregulated in response to parasite infection. *T*. *cruzi* amastigotes harvested from DGAT1/2^-/-^ fibroblasts incorporated less ^14^C-label into neutral lipid (Fig 6D) and phospholipid (Fig 6E) classes as compared to the same number of parasites derived from WT fibroblasts or from DGAT2-complemented DGAT1/2^-/-^ fibroblasts (Fig 6D and 6E). Given that the DGAT1/2^-/-^ cells were not generally impaired in ^14^C-palmitate uptake and incorporation into other neutral lipid or phospholipid classes (Fig 6D and 6E), our results indicate that host DGAT-dependent TG synthesis is a major route of LCFA acquisition by *T*. *cruzi* amastigotes. Thus, despite increased TG labeling in parasite-infected DGAT1/2-/- cells our results suggest that the non-DGAT-derived TG pool may be inaccessible to the parasite.

We sought to determine the impact of decreased access to host FA through the TG pool on amastigote growth. Using a flow cytometry-based method to follow amastigote proliferation using CFSE-labeled parasites [22] we find that *T*. *cruzi* amastigotes undergo fewer divisions in DGAT1/2^-/-^ cells as compared to WT MEF or in DGAT2-complemented DGAT1/2^-/-^ fibroblasts (Fig 6F). These combined lipidomic, metabolic labeling, and proliferation data suggest that *T*. *cruzi* amastigotes are capable of scavenging LCFA primarily from the host GL pool and that, specifically, the host TG pool is important for maintaining maximal parasite growth. However, amastigotes are still able to proliferate in host cells lacking the capacity for TG synthesis via the major DGAT-dependent pathway [37]. Such predicted metabolic flexibility may be due to the capacity for parasites to access TG generated via PC in DGAT-independent manner (discussed in [37]) or to synthesize their own LCFA *de novo* [25] via a sequential FA elongase system [38], and/or parasite access to host FA independent of the host TG pool.

## Discussion

Metabolic coupling to host cellular and biochemical pathways is universally required to sustain the growth and/or survival of obligate intracellular pathogens. In addition to acquiring essential nutrients from their host cells, intracellular pathogens often scavenge metabolites and/or macromolecules that they have the intrinsic capacity to synthesize [39, 40]. This strategy may be more energetically favorable and offer a level of flexibility that can facilitate pathogen survival under changing environmental conditions. Here, we demonstrate that, despite the predicted capacity for *de novo* FA synthesis by the kinetoplastid protozoan parasite, *Trypanosoma cruzi* [31], the obligate intracellular amastigote stages of this parasite readily incorporate long-chain fatty acids (LCFA), acquired from mammalian host cell glycerolipid (GL) pools, into their own lipid storage and synthesis pathways. Our findings expose a biochemical and functional link between parasite and host lipid metabolism and demonstrate the potential or *T*. *cruzi* to bypass its own FA and lipid biosynthetic capabilities predicted in expression analyses [23–25].

The unbiased, quantitative lipidomics approach adopted in this study, which focused on FA moiety compositions in different parasite and host cell lipid subclasses, was instrumental in revealing the hybrid nature of the *T*. *cruzi* lipidome. A key element of our study design was the generation of parallel comprehensive lipidomic datasets for *T*. *cruzi* amastigotes isolated from two distinct mammalian host cell types and the subsequent comparisons made between parasites and their cognate host cells. A deep analysis of the FA moiety distribution within each lipid subclass was conducted with extensive manual curation to facilitate the high confidence assignment of host- and parasite-like lipid signatures. Overall, we find that *T*. *cruzi* amastigotes maintain a lipid identity that is distinguishable from that of their mammalian host cells. First, the proportion of several major lipid classes identified in *T*. *cruzi* amastigotes remained constant over four independent experiments and was shown to be independent of the host cell type that housed the parasite, despite overt differences between fibroblasts and myoblasts. Additionally, *T*. *cruzi* amastigotes display strong parasite-specific signatures within the GP pool with lipids enriched in C18:2, a FA moiety that is not synthesized by mammalian cells, but differentially produced by *T*. *cruzi* via the action of an oleate delta-12 desaturase [32]. This parasite also contains relatively high levels of ether-bound GP and proportionally high levels of VLC-PUFA in their PC pools, as compared to mammalian host cells. Such conservation of class-specific lipid moiety distribution in *T*. *cruzi* amastigotes suggests that these molecules play important biological roles in this organism, including differentiation, cell signaling, and modulation of host immune responses, as has been shown for the specific LPC distribution of *T*. *cruzi* trypomastigotes and amastigotes [41, 42].

Another consistent feature among parasites isolated from different mammalian cell types was the relative enrichment of TG, a main storage lipid in cells, which comprised approximately 25% of the *T*. *cruzi* amastigote lipidome. However, unlike the parasite signatures noted above, the *T*. *cruzi* TG and DG pools, along with the LCFA-containing PC pool, displayed a strong host signature. In fact, the FA moiety profiles for these lipid subclasses were nearly identical to their specific host cell counterparts, suggesting that these lipids were acquired by the parasites from their mammalian host cells. This conclusion is supported by metabolic labeling studies in which incorporation of exogenous FA into amastigote neutral lipids and phospholipids involves flux through host TG pools. Exogenous provision of either odd-chain FA (C15:0) or radiolabeled FA (C16:0) resulted in their incorporation into a range of parasite-associated neutral lipids and phospholipids. However, trafficking of these exogenous FA tracers into host-resident *T*. *cruzi* amastigotes was significantly diminished in parasites that were grown in fibroblasts lacking enzymes required for *de novo* TG synthesis, DGAT1/2 [37], this trafficking into amastigote was restored upon genetic complementation with DGAT2. Furthermore, as the proliferative capacity of *T*. *cruzi* ICA was significantly reduced in DGAT1/2-deficient fibroblasts and restored with ectopic expression of DGAT2, we conclude that host TG pools likely serve as an important source of FA needed for different biological activities in the parasite, including lipid and membrane synthesis, β-oxidation, and the generation of bioactive lipid mediators [23–25, 43].

Together, our observations support a model in which intracellular *T*. *cruzi* amastigotes assemble a mosaic lipidome that combines scavenged and *de novo* synthesized FA. Scavenged lipids may serve as a source of FA during the proliferative phase of the *T*. *cruzi* intracellular lifecycle and/or during the intracellular conversion of amastigotes to trypomastigotes, a time when extensive lipid remodeling is anticipated [44]. Evidence that host-derived TG/DG are utilized, at least in part, for the synthesis of parasite phosphatidylinositols (PI) was seen in the metabolic incorporation of exogenous FA into parasite PI in a DGAT1/2-dependent manner. In contrast, exogenous FA were not detectably incorporated into *T*. *cruzi* PE, a relatively abundant lipid class, suggesting a route of PE synthesis intrinsic to the parasite. Since the bulk of PE in *T*. *cruzi* amastigote was shown to be ether-bound (plasmalogen), it is possible that the lack of labeling of these lipids with exogenous FA is a consequence of the compartmentalized biosynthesis of ether lipids in the parasite glycosomes (organelles which are roughly equivalent to mammalian peroxisomes) [45]. The lack of flux of exogenous FA into parasite glycosomes would also explain the relatively low labeling of the *T*. *cruzi* PI pools, which were also shown to be rich in ether-bound species. While more detailed metabolic flux analyses are required to fully appreciate the contribution of scavenged FA to the biology of intracellular *T*. *cruzi* amastigotes, these data suggest that pathways involved in the synthesis of certain parasite lipids may not converge with the exogenously supplied FA obtained from host TG pools. As such, *T*. *cruzi* amastigotes may rely on their own FA synthesis capacity to generate a subset of lipids, which may be subject to differential regulation. *T*. *cruzi* exploits an uncommon modular synthesis pathway shared with other kinetoplastids to synthesize LCFA *de novo*, that relies on FA elongases instead of type I or type II FA synthases typically found in other organisms [31, 46]. This modular synthesis involves 3 elongases (ELO 1–3), which convert C4:0 to C10:0 (ELO1), C10:0 to C14:0 (ELO 2), and C14:0 to C18:0 (ELO3). A fourth elongase (ELO4) is responsible for the synthesis of very-long chain fatty acids (VLCFA), possibly including VLC-PUFA. Although ELO1-3 are highly expressed in host cell resident *T*. *cruzi* amastigotes [25], it is currently unknown whether these enzymes are active in this life cycle stage [25] or essential. Based on our current finding that *T*. *cruzi* amastigotes scavenge and utilize host lipid-derived FA for the synthesis of certain lipids, but not others, we predict that the *T*. *cruzi* FA elongase system may be required for this life cycle stage of the parasite. In addition to generating FA *de novo*, *T*. *cruzi* FA elongases, along with parasite desaturases, are likely to be used to modify scavenged LCFA before incorporation into parasite lipids. The relative reliance of *T*. *cruzi* on exogenous versus endogenous FA, and whether environmental conditions alter this balance, remains to be determined.

Our data strongly suggest that *T*. *cruzi* amastigotes scavenge TG, DG, and LCFA-PC from their mammalian host cells during intracellular infection. As the bulk of the cellular TG pool is sequestered in lipid droplets (LD), that are comprised of a neutral lipid core (TG and DG) surrounded by a phospholipid monolayer enriched in saturated LCFA-PC [47], we propose that TG, DG and LCFA-PC are acquired *en masse* from host lipid droplets. The alternative model, in which FA stored in host TG/DG pools are mobilized through the action of TG lipases [48], and then taken up by the parasite and reassembled into TG, DG and LCFA-PC with the same FA moiety distribution as existed in the host cell, is unlikely. As lipid droplets are highly dynamic organelles that function as critical hubs for FA trafficking in cells with key roles in cellular lipid and energy metabolism [49–51], it is not surprising that host LD are frequently targeted by intracellular pathogens. LD accumulation is a common cellular response to pathogen infection [52–55], which can occur in response to increased oxidative stress [56, 57] or paracrine signals [58]. With high rates of FA flux, host cell LD represent a readily accessible source of FA for a number of intracellular pathogens, such as *Chlamydia trachomatis*, *Mycobacterium tuberculosis*, and *M*. *leprae*, that exploit host LD to obtain lipids for energy and membrane biosynthesis [59]. Our demonstration of a biochemical interaction between *T*. *cruzi* amastigotes and host TG, which are mainly found in lipid droplets, brings functional insight to earlier descriptions of increased LD content in *T*. *cruzi* infected macrophages [60] and the clustering of intracellular parasites in the vicinity of host LD in adipocytes, macrophages, and cardiomyocytes [15, 61]. During acute Chagas disease, inflammatory macrophages typically exhibit increased formation of LD enriched with arachidonic acid (AA), which is a precursor for the synthesis of proinflammatory eicosanoids such as prostaglandin E_2_ [61]. Moreover, these LD have been shown to contain eicosanoid-forming enzymes (cyclooxygenases and lipoxygenases) that are upregulated during *T*. *cruzi* infection [61]. Despite the importance of LD for the storage of AA in inflammatory macrophages and other leukocytes [62, 63], we were unable to detect appreciable levels of this FA in the TG pools of either infected or mock-infected host cells, or *T*. *cruzi* amastigotes. On the contrary, most of the esterified AA was identified in the PE and PI pools of host cells, while *T*. cruzi amastigotes had consistently low levels of this FA throughout its lipidome. These differences likely reflect the substantial variation in lipid droplet composition and function between cell types, and even within a homogenous cell population under different environmental conditions [64].

In summary, the application of a comparative lipidomics approach successfully distinguished parasite- and host-specific lipidomic signatures, providing evidence that *T*. *cruzi* amastigotes acquire a substantial portion of their lipidome from host TG pools, possibly via the direct acquisition of host lipid droplets. With this strategy, we show that future research on TG and LD metabolism in the context of *T*. *cruzi* infection is predicted to yield important information pertaining to the mechanisms of *T*. *cruzi* persistence and recrudescence during Chagas disease.

## Methods

### Mammalian cell culture and *T*. *cruzi* maintenance

Mammalian cell lines: human foreskin fibroblast (HFF; provided by S. Lourido, MIT), mouse skeletal muscle myoblast (C2C12; ATCC CRL-1772), African green monkey kidney epithelial (LLcMK2; ATCC CCL-7), mouse embryonic fibroblast (MEF), and diacylglycerol acyltransferase 1/2-deficient MEF (DGAT1/2^-/-^; generously provided by the Walther-Farese laboratory at Harvard T. H. Chan School of Public Health [37]). DGAT1/2^-/-^ complementation with OriGene Mouse cDNA ORF Clone of *Dgat2* (NM_026384) was performed according with the manufacturer’s protocol for the generation of stable transfectants. Mammalian cells were maintained in Dulbecco’s Modified Eagle Medium (DMEM) supplemented with 1mM pyruvate, 25 mM glucose, 2 mM glutamine, 100 U/ml penicillin, 10 μg/ml streptomycin and 10% fetal bovine serum (FBS) (DMEM-10). Cell culture reagents were purchased from Gibco. *Trypanosoma cruzi* Tulahuén strain parasites (ATCC PRA-33) were selected because of their frequent use in high-throughput screens for novel anti-trypanosomal drug discovery and functional studies [65] and [22, 66]. Parasites were maintained by weekly passage in LLcMK2 cells in DMEM supplemented with 1 mM pyruvate, 25 mM glucose, 2 mM glutamine, 100 U/ml penicillin, 10 μg/ml streptomycin and 2% FBS (DMEM-2) at 37°C, 5% CO_2_ as previously described [22, 66]. Motile extracellular trypomastigotes were collected from infected LLcMK2 supernatants, pelleted at 2,000*g* for 10 min and allowed to swim up from the pellet for a minimum of 2 h at 37°C, 5% CO_2_ before collection and use for experimental infection.

### Experimental *T*. *cruzi* infection and intracellular amastigote isolation

HFF, C2C12, or MEF, as indicated, were seeded in T-75 flasks and grown to 80% confluence over 2 days in DMEM-10. To establish intracellular *T*. *cruzi* infection, host cell monolayers were incubated with 1 x 10^7^ tissue culture-derived trypomastigotes (TCT) for 2 h at 37°C, 5% CO_2_ in DMEM-2. Cells were then rinsed twice with PBS to remove extracellular parasites and fresh DMEM-2 medium added to flasks and incubated for a further 46 h. Parallel cultures of mock-infected mammalian cell monolayers were also established. The isolation of the intracellular amastigotes (ICA) form of *T*. *cruzi*, was conducted using a protocol modified from [67]. Briefly, infected monolayers were washed extensively with PBS, detached from the flask using mild trypsinization (Gibco, 0.05% Trypsin-EDTA), resuspended in DMEM-2 and pelleted by centrifugation at 300*g* for 10 m. After aspirating the supernatant, cells were washed with cytosolic buffer (10 mM NaCl, 140 mM KCl, 2 mM MgCl_2_, 2 μM CaCl_2_, 10 mM HEPES, pH 7.4 [68] and suspended in 10 ml cytosolic buffer. Of this infected cell suspension, 0.5 ml was retained as the “infected” sample and the remaining 9.5 ml was subjected to 2 rounds of cell disruption using a Miltenyi GentleMACS dissociator (M tubes, Protein_01 protocol) and host cell lysis was visually confirmed. The lysate was then passed over a 4 ml DEAE-Sephacel (Sigma) column (pre-washed with 10 column volumes cytosolic buffer), and cytosolic buffer was added such that three 10 ml flow-through fractions could be collected. Isolated amastigote from each fraction were enumerated by haemocytometer and fractions containing *T*. *cruzi* amastigotes were pooled, pelleted, decanted, and then frozen at -80°C until analysis.

### Protein quantitation

Quantitation of protein content in host cell and parasite lysates was determined using the ThermoPierce BCA assay reagent kit as per manufacturer’s instructions for microplate assay, using bovine serum albumin as the reference standard.

### Western blotting

Host cell or parasite lysates were adjusted to 2 μg/μl in 2X Laemmli sample buffer containing 100mM β-mercaptoethanol, heated to 95°C for 3 min in and 10 μl (20 μg protein) and separated by polyacrylamide gel electrophoresis on 4–20% Bio-Rad Mini-PROTEAN TGX Precast Gels and transferred to Immobilon-FL 0.45 μm PVDF membrane by semi-dry transfer (Bio-Rad Trans-Blot SD Semi-Dry Transfer Cell) for 30 min at 20V. Membranes were blocked for 1 h at ambient temperature with shaking in 1:1 mixture of SEA BLOCK Blocking Buffer (Thermo Pierce) and phosphate-buffered saline. Primary antibodies were incubated for 16 h at 4°C at the following dilutions, Abcam ATPB antibody [3D5], 1:2000; Cell Signaling IRE1 alpha [14C10], 1:1000; Novus Biologicals Perilipin-3/TIP47 antibody, 1:1000; Sigma FLAG, 1:500. All secondary antibodies were incubated for 30 m at room temperature at the following conditions AlexaFluor (647 goat anti-mouse) 1:10,000; Donkey anti-rabbit DyLight 800 Thermo Fisher 1:10:000. All antibodies were diluted with a 1:1 mixture of SEA BLOCK Blocking Buffer and phosphate-buffered saline containing 0.2% Tween 20. Membranes were imaged using the Odyssey Infrared Imaging System (LI-COR Biosciences).

### Lipid extraction

All solvents used were of HPLC grade or higher. The lipid extraction protocol was modified from [42]. Briefly, cell lysates (biological replicates, containing the following standard lipid mix per 100 μg protein lysate: 375 pmoles C17:1 lysophosphatidic acid; 225 pmoles C17:0/C20:4 phosphatidic acid; 170 pmoles C17:1 lysophosphatidylserine; 180 pmoles C17:0/C20:4 phosphatidylserine; 86 pmoles C17:1 lysophosphatidylethanolamine; 112 pmoles C17:0/C14:1 phosphatidylethanolamine; 95 pmoles C17:1 lysophosphatidylcholine; 112 pmoles C17:0/C20:4 phosphatidylcholine; 33.2 pmoles C17:1 lysophosphatidylinositol; 165 pmoles C17:0/C20:4 phosphatidylinositol; 105 pmoles C17:0/C14:1 phosphatidylglycerol; 180 pmoles C17:0/d18:1 ceramide; 140 pmoles C17:0/d18:1 sphingomyelin; 155 pmoles C12:0/d18:1 β-glucosyl-ceramide; and 60 pmoles C17:1/C17:1/C17:1 triacylglycerol) were suspended in ice-cold HPLC-grade water, and transferred to 13x100-mm Pyrex tubes with polytetrafluoroethylene (PTFE)-lined screw caps. HPLC-grade water, methanol, and chloroform were added to a final chloroform/methanol/water (C/M/W) ratio of 1:2:0.8 (v/v/v). Samples were vortexed vigorously for 5 min, and centrifuged for 10 min at 1,800*g* at room temperature. After centrifugation, the supernatants were transferred to new Pyrex tubes, and the pellets were dried under N_2_ stream. The pellets were then extracted with chloroform/methanol (2:1, v/v), centrifuged, and the resulting supernatants were combined with the corresponding supernatants from the first step of extraction (C/M/W 1:2:0.8 v/v/v), and dried under N_2_ stream. Samples were then subjected to Folch partitioning [69] by dissolving them in C/M/W (4:2:1.5, v/v/v), followed by vortexing and centrifuging, as described above. After centrifugation, the lower (organic) and upper (aqueous) phases were separated into fresh Pyrex tubes. The aqueous upper phase was then re-extracted with C/M (2:1 v/v), and the resulting organic phase was combined with the organic phase from the preceding step. The pooled organic phases were dried under N_2_ stream and stored at -20°C until analysis.

### UHPLC-ESI-MS/MS for lipidomics

Extracted lipid samples were diluted in 50 μl of C/M (2:1 v/v) and analyzed by UHPLC-ESI-MS, method modified from [70]. UHPLC-ESI-MS/MS was conducted using a Dionex UltiMate 3000 UHPLC system (Thermo Scientific) coupled to a Q Exactive Hybrid Quadrupole-Orbitrap Mass Spectrometer (Thermo Scientific). 5 μl of each sample was injected onto an Accucore (Thermo Scientific) C18 LC column (2.1 mm x 150 mm x 2.6 μm particle size). Lipids were fractionated by reverse-phase chromatography over a 46 min gradient (mobile phase A: acetonitrile/water (50:50 v/v), 10 mM ammonium formate, 0.2% formic acid; mobile phase B: methanol/isopropanol/water (10:88:20 v/v/v), 2 mM ammonium formate, 0.01% formic acid), with the following program, constant flow rate of 500 μL/min, 35–45% B over 0–5 min, 45–85% B from 5–28 min, 85–100% B from 28–38 min, followed by an immediate drop to 35% B, held constant up to min 46. The column temperature was set to 50°C, and the autosampler tray temperature was set to 10°C. The HESI II ion source (Thermo Scientific) was set as follows: sheath gas flow rate = 60; auxiliary flow rate = 20; sweep gas flow rate = 1; spray voltage (KV) = 3.00; capillary temperature = 285°C, S-Lens RF level = 45, and auxiliary temperature = 370°C.

The mass spectrometer acquisition settings were as follows for both positive and negative ionization mode: Full Scan–top 15 data-dependent MS/MS. Full scan was set for a range of 250–1800 m/z. The mass resolution was set to 70,000; AGC target was set to 1e6, the C-trap ion accumulation time was set to 120 ms; data dependent MS/MS was set to a mass resolution of 30,000, AGC target was set to 5e5, the C-trap ion accumulation time was set to 120 ms, select ion exclusion was set to 8 s, and the HCD (higher-energy collisional dissociation) fragmentation ramp was set to 15, 25, and 35 NCE (normalized collision energy). All data were analyzed using LipidSearch Software Version 4.2 (Thermo Scientific) and all identified species (A, B and C quality) were validated manually as detailed in Supporting Information: Supplemental Methods.

### Calculation of lipid class fatty acid composition

Within a lipid class, MainArea values output by LipidSearch were assigned to each FA moiety of a given species and summed using Microsoft Excel PivotTable. Area % is the summed FA value divided by the lipid class total, multiplied by 100 to reflect percentage.

### Determination of lipid class distribution

Known quantities of each lipid standard were analyzed using the same UHPLC-ESI-MS/MS methods described below to generate a response factor (RF; peak area/pmol standard injected). The RF for each standard was divided by the CerG1 RF to calculate a molar relative response factor (MRRF) for each major lipid class. The MRRF for each class was normalized to the CerG1 peak area of each sample, and peak areas adjusted accordingly. Only those classes for which standards were detected in each run were considered for analysis. Each pie chart is the average of at least 3 biological replicates.

### [^14^C(U)]-palmitate incorporation and thin layer chromatography

Mock- and *T*. *cruzi*-infected mammalian cell monolayers were incubated with DMEM-2 supplemented with 0.3 μCi/ml [^14^C(U)]-palmitate (Perkin Elmer) and 25 nM palmitate (Cayman Chemical) [71] for 6 h and monolayers washed extensively in PBS to remove unincorporated label. *T*. *cruzi* amastigotes were isolated from infected cells and lipids were extracted from equivalent protein amounts of all labeled samples as described above, without inclusion of internal standards, and lipid extract equivalent to 10 μg protein per sample was subjected to thin layer chromatography (TLC).

#### Neutral lipid TLC

Neutral lipid TLC were conducted using a protocol modified from [71]. Briefly, glass-backed silica plates (Silica gel 60 20 cm x 20 cm, Millipore) were preconditioned with hexane, air-dried, and baked at 100°C for 30 min. The TLC chamber was equilibrated with hexane: diethyl ether: acetic acid (80: 20: 3 v/v/v) for at least 2 h prior to developing. The identity of different lipid classes on the TLC plate was inferred by comparing their migration pattern to that of commercial standards run on the same plate. The following standards were used for neutral lipid analysis (5 μg each): cholesterol, palmitic acid, cholesteryl-palmitate, 1,3-diacylglycerol (1-Palmitoyl-3-stearoyl-rac-glycerol, Sigma-Aldrich), 1,2-diacylglycerol (C18:1/C18:1-DAG), and C18:1/C18:1/C18:1-triacylglycerol (triolein, Avanti Polar Lipids).

#### Glycerophospholipid TLC

Phospholipid TLC were conducted using a protocol modified from [72]. Briefly, glass-backed silica plates (Silica gel 60 20 cm x 20 cm, Millipore) plates were preconditioned with dichloromethane, dried, and baked at 100°C for 30 min. The TLC chamber was equilibrated with acetone: methanol: acetic acid: chloroform: water (30: 26: 24: 80: 10 v/v/v/v/v) for at least 2 h prior to developing. The following standards were used for glycerophospholipid analysis (10 μg): bovine brain phosphatidylserine, egg phosphatidylglycerol, heart cardiolipin (Avanti Polar Lipids); soy phosphatidylcholine, brain phosphatidylethanolamine, soy phosphatidylinositol, egg yolk phosphatidic acid, bovine brain L-α-Lysophosphatidylcholine, and egg yolk 3-sn-Lysophosphatidylethanolamine (Sigma-Aldrich).

Once developed, TLC plates were dried, covered with mylar, and exposed to a storage phosphorimager screen overnight. Fluorescence was visualized using a Typhoon FLA 9500 Scanner (GE Healthcare). After imaging, TLC plates were exposed to iodine vapor to visualize equal loading.

### Data analysis, visualization, and interpretation

The web-based server MetaboAnalyst 3.0 was used to perform principle component analyses (PCA) on median normalized, and log transformed species abundances [73]. Bar and pie charts were generated using GraphPad Prism 7, version 7.0b for Mac OSX.

### Quantification of amastigote proliferation by flow cytometry

Trypomastigotes were diluted to 5x10^6^ parasites/mL in PBS and stained with a final concentration of 1μM carboxyfluorescein succinimidyl ester (CFSE) (Thermo Fisher) for 15 min at 37°C. After staining, trypomastigotes were washed and re-suspended in DMEM-2 and allowed to infect host cells as described. At various time points after infection amastigotes were isolated and fixed on ice in 4% paraformaldehyde/PBS for 20 min. After fixation amastigotes were centrifuged at 4,000*g* for 10 min and the resulting pellet was resuspended in PBS and kept at 4°C until preparation for acquisition. Immediately prior to acquisition amastigotes were pelleted at 4,000*g* for 10 min and resuspended in a 0.1% Triton X-100/PBS permeabilization solution containing 10 ng/mL DAPI (Sigma-Aldrich) for a minimum of 30 min on ice. Events were acquired using a LSR II (Becton Dickinson). Amastigotes were identified based on size and DAPI staining. Proliferation modeling based on signal intensity from undivided parasites collected at 18 hpi were generated using FlowJo (Tree Star) proliferation software. Greater than 10,000 events in the final amastigote gate were acquired for each sample.

## Supporting information

S1 Methods Detailed supplemental methods with corresponding references are included in file. (DOCX)

S1 Fig Negative ion mode base peak chromatograms of *T*. *cruzi* amastigotes and host cells. Negative ion mode base peak chromatogram of C2C12, HFF, and cognate *T*. *cruzi* amastigote (cICA and hICA, respectively). The major lipid subclasses eluting at different retention times (min) are indicated above the chromatogram. (TG–triacylglycerol, DG–diacylglycerol, Cer–ceramide, CerG–hexosylceramide, SM–sphingomyelin, LPC–lysophosphatidylcholine, PC–phosphatidylcholine, LPE–lysophosphatidylethanolamine, PE–phosphatidylethanolamine, LPS–lysophosphatidylserine, PS–phosphatidylserine, PI–phosphatidylinositol, PG–phosphatidylglycerol).
(TIF)

S2 Fig Principle component analysis of host and parasite lipidomes at the lipid species level. Principle component analysis of lipid species are plotted for **(A)** PC, **(B)** PE, and **(C)** PS subclasses. The first two principle components are plotted (PC1 and PC2) with proportion of variance for each component shown in parenthesis. Each sample is represented and the 95% confidence interval indicated in shaded circle.
(TIF)

S3 Fig Gas chromatography with flame ionization detection (GC-FID) analysis of esterified FA from the total lipid pool. Esterified FA from lipid extracts were hydrolyzed with ammonium hydroxide at 37°C for 1 h, and methylated with 0.5 N methanolic-HCl for 1 h at 85°C. Methylated FA were recovered by dichloromethane:water partitioning and analyzed by GC-FID. Area % is plotted for each FA moiety detected. Data are from a representative experiment.
(TIF)

S4 Fig Trends in host and parasite FA composition varies between lipid classes. FA area % is plotted for long-chain fatty acid (LCFA) and very long-chain polyunsaturated fatty acid (VLC-PUFA) of (A) PC, (B) LPC, (C) PE and (D) PI for samples derived from C2C12 host cells (HFF main text, Fig 4). Data are represented as mean ± standard deviation.
(TIF)

S5 Fig FA composition in TG and DG of *T*. *cruzi* intracellular amastigotes mirrors host cells. FA area % is plotted for (A) TG and (B) DG classes for samples derived from C2C12 host cells; (HFF main text plotted in Fig 5). Data are represented as mean ± standard deviation.
(TIF)

S6 Fig Immunoblot confirmation of ectopically expressed DGAT2. Protein expression of Myc-DDK-DGAT2 was confirmed by immunoblotting using the FLAG M2 antibody.
(TIF)

S7 Fig Annotated negative ion mode MS/MS spectrum of *T*. *cruzi* IPC (34:1). Representative MS/MS spectrum of the most abundant IPC species identified in *T*. *cruzi* was acquired from lipid extracts of TCT as described in the supplementary methods section of the manuscript (Supporting Information: Supplemental Methods). Fragment ions from MS/MS analysis are indicated in the figure, according to previously published data [74].
(TIF)

S1 Table Summary of unique lipid species, by class, identified using LC-MS/MS. (XLSX)

S2 Table Detection of lipid species across all samples. Summary of the lipid species detected in at least one biological replicate of each sample. An ‘x’ indicates the species was detected, ND = not detected.
(XLSX)

S3 Table Summary of lipid class breakdown in *T*. *cruzi* and mammalian cells. The relative abundance of the major lipid subclasses of mammalian host cells (C2C12 and HFF) and *T*. *cruzi* intracellular amastigotes (ICA) and tissue-culture trypomastigotes (TCT) represented as a portion of total lipid content in each sample, averaged for 4 independent experiments. TG–triacylglycerol, DG–diacylglycerol, Cer–ceramide, CerG–hexosylceramide, SM–sphingomyelin, PC–phosphatidylcholine, PE–phosphatidylethanolamine, PS–phosphatidylserine, PI–phosphatidylinositol, PG–phosphatidylglycerol.
(XLSX)

S4 Table Total lipidome fatty acyl composition by average area percent. Area % was determined for each FA in every lipid class. Averages from all lipid classes are shown.
(XLSX)
