# Supplementary material for: Host triacylglycerols shape the lipidome of intracellular trypanosomes and modulate their growth
Source: PLoS Pathog. 2017 Dec 27;13(12):e1006800. doi: 10.1371/journal.ppat.1006800 (PMC5760102; doi:10.1371/journal.ppat.1006800)
Supplement: S1 Methods — (DOCX) [file ppat.1006800.s001.docx]

**Supporting information**

**Identification of lipid species using LipidSearch^TM^ Software (Thermo Fisher Scientific)**

Raw files generated by the LC-MS/MS runs were analyzed using the LipidSearch^TM^ Software for the identification of lipid species, combining accurate-mass information from parent ion with MS2 information. The following parameters were used for batch analysis: Database – Q Exactive; Search Type – Product; Experiment Type (Exp Type) - LC-MS; Parent Tolerance (Parent Tol) - 0.1 Da; NL/Prec Tol – 0.5 Da; Precursor Tolerance (Prec Tol) - 10.0 ppm; Product Tolerance (15.0) ppm; Merge Range (Min) - 0.0; Minimal Peak Width (min) - 0.0; Threshold Type - Relative; Product Ion – 1.0%; m-score threshold – 2.0; Recalculate Isotope – ON; R.T. Interval (min) - 0.01; Execute Quantitation – ON; *m/z* Tolerance (*m/z* tol) - -10.0 / + 10.0; Tolerance Type – ppm; R.T. range (min) - -0.05 / +0.05; Toprank filter – ON; Main Node Filter – Main Isomer Peak; m-Score Threshold (Display) - 5.0; C-Score Threshold (Display) - 2.0; Fatty Acid Priority (FA Priority) - ON; ID Quality Filter (A; B; C). The following parameters were used for sample alignment: Search Type – Product; Experiment Type (ExpType) - LC – MS; Alignment Method – Max; R.T. Tolerance – 0.25; Calculate Unassigned Peak Area – ON; Filter Type – New Filter; Toprank Filter –ON; Main Node Filter – Main Isomer Peak; m-score threshold – 5.0; ID Quality – A,B,C.

Because this study required a careful comparison between the lipid composition of *T. cruzi* and its host cells, all lipid species identified using the LipidSearch^TM^ software were manually curated after computational analysis, being either accepted, rejected, or reassigned, based on the parameters described below.

**Curation of MS/MS spectra**

**Inositol PL composition:** A total of 34 PI molecular species were detected in negative ion mode as [M–H]^−^ ions in a calculated mass range of 598.3 – 840.5 a.m.u. The major fragment ions used for the structural assignment of diacyl PI molecular species were the characteristic peak at *m/z* 241, corresponding to a *myo-*inositol-1,2-cyclic phosphate anion, as well as prominent peaks corresponding to FA anions. Other informative peaks included *lyso*-PIs, arising from the loss of a FA moiety from the parent ion, and *lyso*-PAs, generated from the loss of a FA moiety and a dehydrated inositol from the parent ion or from the loss of a dehydrated inositol from *lyso*-PI. Ether-linked molecular species of PIs were also identified based on the presence of the peak at *m/z* 241, and of a peak corresponding to the FA anion arising from the loss of the FA moiety linked to the *sn*-2 region of the phosphoglycerol backbone. The presence of the ether-linked fatty alcohol moiety at the *sn-*1 position of the phosphoglycerol backbone was inferred by the presence of prominent fragment ions corresponding to ether-linked *lyso*-PIs, dehydrated ether-linked *lyso*-PIs, ether-linked *lyso*-PA, and to dehydrated ether-linked *lyso*-PA. All ether-linked PI species characterized in this study had the same 16:0e fatty alcohol moiety (hexadecanol), with structural diversity arising from the presence of a different FA moiety attached to the *sn-*2 position of each molecular species. Despite high levels of ether-type PL having been reported for other trypanosomatids, such as *T. brucei* [1, 2], *T. congolense* (reviewed in [3])*,* and *Leishmania spp.* (reviewed in [4]), a high abundance of of 16:0e/18:1- and 16:0e/18:2-PI in *T. cruzi* ICA, hasn’t been reported in other parasites (Figs 4A and 4B, S1 Table). The presence of these species is likely associated with the fact that *T. cruzi* expresses high levels of ether-bound glycoinositolphospholipids (GIPLs) and of glycosylphosphatidylinositol-anchored proteins (GPI-AP) on its surface [5].

**Choline PL composition:** A total of 380 PC molecular species were detected in either positive ion or negative ion mode in a calculated mass range of 495.3 – 1041.9 a.m.u. The majority of PC molecular species were identified in positive ion mode, forming either [M+H]^+^ or [M+Na]^+^ cations. Although very abundant, [M+H]^+^ ions provided very little structural information of PC molecular species, as described before. In agreement with extensive literature on the subject, the major fragment ion observed upon fragmentation of [M+H]^+^ diacyl-PCs was the ion at *m/z* 184, which corresponds to a protonated phosphocholine, while fragment ions containing structural information pertaining to the FA moieties of individual PC molecular species were not detected. Under these circumstances, the identity of the specific FA moieties present in each PC molecular species could not be determined. In these cases, our results are reported in the x:y form, where “x” represents the total number of FA carbons (sum of carbon atoms of the two FA moieties) and “y” represents the total number of double bonds (sum of double bonds of the two FA moieties) in a given molecular species, as calculated based on the high mass-accuracy of our analyses. Because it is likely that diacyl-PCs containing different FA moieties might have the same total number of carbon atoms and double bonds, our conclusions pertaining to the distribution of diacyl-PC species containing the “x:y” formula described above assume that every species having a given x:y formula probably consist of several unique diacyl-PC molecular species that happen to have the same number of carbons and double bonds. The same “x:y” notation was also applied to molecular species belonging to other lipid subclasses when the identity of their individual FA substituents could not be determined. The MS/MS analysis of [M+Na]^+^ ions, however, gave rise to more informative structural information than [M+H]^+^ions. The fragmentation of [M+Na]^+^ ions yielded abundant fragment ions corresponding to the loss of a trimethylamine from the phosphocholine headgroup ([M+Na – N(CH_3_)_3_]^+^), as well as ions corresponding to the neutral loss of a FA moiety in conjunction with a trimethylamine. The identity of the FA moieties is further corroborated by the presence of fragment ions corresponding to dehydrated sodiated and to dehydrated protonated LPCs, arising from the neutral loss of one of the FA moieties from the parent ion. Finally, two abundant fragment ions corresponding to the neutral loss of either a sodiated or of a protonated phosphocholine headgroup corroborate the identity of the headgroup. Whenever possible, the identity of the FA moieties present in individual diacyl-PC molecular species was corroborated through the analysis of ions formed with formate adducts in the negative ion mode. The main fragment ions observed through the MS/MS analysis of [M + HCOO]^-^ ions consisted of the two FA anions originated from the parent ion, as well as the well-documented [M – CH_3_ – HCOO]^-^ ion obtained through the loss of a methyl group from the phosphocholine headgroup, along with the formate adduct. The identity of the FA moieties was further verified through the presence of two [LPC - CH_3_]^-^ ions through the loss of one of the FA moieties and of a methyl group from the parent ion. Despite there being abundant literature demonstrating that a careful analysis of the relative abundance of fragment ions generated from the MS/MS fragmentation of diacyl-PCs forming adducts with alkali metals (usually Na^+^ or Li^+^) in the positive ion mode or forming adducts with formate in the negative ion mode can be used to establish the regiospecific location of fatty acyl moieties in each diacyl-PC molecular species, we did not conduct any experiments using the appropriate set of lipid standards to be able to make this type of distinction using the data we report in this work. Therefore, our conclusions pertaining to the FA distribution in diacyl-PCs are based on the relative abundance of species containing certain FA moieties, as well as the relative abundance of different fatty acyl moieties within the diacyl-PC pool, but not on their regiospecific location.

Aside from diacyl-PCs, 45 species of *lyso*-PC (LPC) were also identified in either positive ion or negative ion mode, in a calculated mass range of 467.3 a.m.u – 719.5 a.m.u. Similar to diacyl-PCs, LPCs were identified primarily in the positive ion mode, forming either [M+H]^+^ or [M+Na]^+^ cations. As was the case for diacyl-PCs, the most abundant fragment ions observed by MS/MS analysis of [M+H]^+^LPC ions corresponded to the protonated phosphocholine at *m/z* 184, and a fragment ion arising from the neutral loss of phosphocholine. In this case, the FA moiety present in each molecular species was calculated based on the exact mass of each parent ion analyzed. As with diacyl-PCs, the MS/MS analysis of [M+Na]^+^ LPC ions yielded richer fragmentation patterns, including the loss of trimethylamine from the sodiated parent ion, and the loss of a sodiated or protonated phosphocholine from the parent ion. Despite being more fragment-rich, the fragmentation profile arising from [M+Na]^+^ cations did not provide more structural information pertaining to the identity of the FA moieties than what was observed for [M+H]^+^adducts. In many cases, the analysis of LPCs forming formate adducts in the negative ion mode generated more informative fragment ions pertaining to the FA moieties of unique LPC lipid species than what could be obtained in positive ion mode. The MS/MS analysis of [M + HCOO]^-^ LPC ions yielded nearly identical fragmentation patterns as described for diacyl-PCs, and the presence of FA anions was used to more accurately determine the identity of each LPC molecular species. In several cases, peaks eluting in two or more retention times in our chromatographic runs were identified to have the same number of carbons and of double bonds. We interpreted that each of these peaks likely corresponded to different regioisomers of LPCs. Using our approach, however, we were unable to determine the precise structure of each of these LPC regiosomers. We therefore reported our findings based on the combined peak areas corresponding to all LPC species having the same number of carbons and double bonds. The same approach was used for other *lyso*-glycerophospholipids.

**Ethanolamine PL composition:** A total of 111 PE molecular species were detected in either positive ion or negative ion mode in a calculated mass range of 527.3 – 941.7 a.m.u. Out of the total number of PE molecular species, 60 belonged to the diacyl-PE subclass, while 51 molecular species belonged to the ether-type subclass (either containing a *O-*1-alkyl or a *O*-1-alkenyl bond). In the negative ion mode, both diacyl-PEs and ether-type PEs were detected forming mostly [M+H]^-^ ions. Upon HCD fragmentation, these parent ions yielded abundant fragment ions corresponding to FA anions. The identity of these FA substituents could be further confirmed through the detection of fragment ions corresponding to negatively charged LPEs. In the case of ether-type PEs, one abundant peak corresponding to the FA moiety dissociated from the *sn*2-position of the phosphoglycerol backbone could be detected, while the identity of the alkyl- or alkenyl-bond fatty alcohol substituent present at the *sn*1 position of the phosphoglycerol backbone could be determined based on the presence of highly abundant ether-type LPE fragment ions. The identity of the headgroup could be determined from the presence of a characteristic fragment ion at *m/z* 140, corresponding to the negatively-charged phosphoethanolamine. In the positive ion mode, the MS/MS analysis of [M+H]^+^ diacyl-PEs and ether-type PEs yielded abundant fragment ions corresponding to the neutral loss of the phosphoethanolamine headgroup (neutral loss of *m/z* 141) from the parent ion. As a trend, *T. cruzi* intracellular amastigotes and trypomastigotes had proportionally higher levels of ether-type PEs than their cognate host cells. These observations agree with previous work that structurally characterized and established the relatively high abundance of two plasmenylethanolamine molecular species in epimastigotes of the Dm28c strain of *T. cruzi* [6]. The two species characterized in their work, namely 16:0p/18:1- and 16:0p/18:2-PE, are also found in high abundance in the two life stages of *T. cruzi* analyzed in our study. These species, however, are found in markedly lower levels in C2C12 and HFF, implying that *T. cruzi* actively maintains high levels of plasmenylethanolamines with oleic or linoleic acid substituents. Because these lipid species were observed using very different approaches, in different parasite strains, as well as in three different life cycle stages, we suggest that they play an important role to the biology of these organisms. We also suggest that these lipids are likely synthesized by these parasites, and not directly scavenged from outside sources, such as the host, or media.

**Serine PL composition:** A total of 54 PS molecular species were identified in either the positive ion mode, forming [M+H]+ cations, or in the negative ion mode, forming in a calculated mass range of 567.3 – 971.7 a.m.u. All the diacyl-PS species identified in the positive ion mode were detected as [M+H]^+^cations. The MS/MS analysis in the positive ion mode of PS molecular species forming [M+H]^+^ ions yielded an abundant fragment ion generated through the neutral loss of the phosphoserine headgroup (loss of *m/z* 185), as well as two fragment ions consisting of dehydroxylated monoacylglycerides. The MS/MS analysis of PS molecular species in the negative ion mode forming [M+H]^-^ ions was used to complement the structural information obtained in the positive ion mode. In the negative ion mode, the identity of the phosphoserine headgroup was confirmed through the identification of fragment ions generated through the neutral loss of the serine moiety (loss of *m/z* 87) from the parent ion, while the identity of the FA substituents was confirmed through the detection of abundant fragment ions corresponding to FA anions. In some cases, fragment ions consisting of the neutral loss of the serine moiety along with one of the FA moieties were also identified, and used to further validate the identity of individual molecular species of PS. Similar to what has been reported in *Leishmania spp* [7, 8] *T. cruzi* amastigote and TCT exhibited proportionally low levels of PS when compared to mammalian cells.

**Cardiolipin composition (CL):** A total of 21 CL species were identified in the negative ion mode as [M – H]^-^ ions in the calculated mass range of 1372 – 1535 a.m.u. The FA moiety composition of CL was readily available from the negative ion mode MS/MS analysis of [M – H]^-^ ions, which yielded abundant fragment ions corresponding to the individual FA anions ([FA – H]^-^) and to the neutral loss of individual fatty acyl substituents from the deprotonated parent ion ([M – FA – H]^-^). The FA composition of *T. cruzi* CL was markedly different from corresponding host cells, corroborating our observations that the amastigote purification procedure used in this study was sufficient to avoid the contamination of these parasites with host-derived mitochondria. Also, this indicates that *T. cruzi* amastigotes are likely not acquiring FA from host CL pools and are synthesizing CL *de novo*.

**Ceramide (Cer), sphingomyelin (SM) and inositol phosphorylceramide composition:** A total of 61 Cer species were identified in a calculated mass range of 509.4 – 661.6 a.m.u, forming mostly [M+H]^+^ adducts in the positive and [M - H]^+^ adducts in the negative ion mode . Individual Cer species were identified by MS/MS in the positive ion mode based on the presence of abundant fragment ion peaks corresponding to either protonated dihydrosphingosine or protonated sphingosine long-chain bases, in combination with the neutral loss of either one or two H_2_O residues ([So – OH]^-^) [9]. The FA moiety for each Cer molecular species could be readily identified in the negative ion mode through the presence of abundant ions corresponding to the dehydroxylated FA moiety ([FA – OH]^-^), as well as ions corresponding to a deprotonated FA moiety ([FA – H]^-^) and to a deprotonated FA amide ([FAmide – H]^-^). In these cases, the structure of the long-chain base was determined through the presence of ions corresponding to the neutral loss of the sphingoid base in conjunction with one or two H_2_O residues. When sufficient structural information was not available, the detailed Cer structure was omitted, and its condensed structure was presented instead.

A total of 76 SM species, in a calculated mass 662.5 – 960.8 a.m.u were identified, forming mostly [M+H]^+^ in the positive ion mode, and [M+HCOO]^+^ in the negative ion mode. The MS/MS analysis of [M+H]^+^ ions yielded mostly an ion at *m/z* 184, corresponding to a protonated phosphocholine, and an ion corresponding to the protonated long-chain base in conjunction with the neutral loss of two H_2_O moieties ([So + H – (H_2_O)_2_]+). The identity of the FA moiety was inferred by calculating its mass based on the parent ion mass and the mass of the long-chain base. Further structural information pertaining to the different SM species was obtained from analyzing the MS/MS fragmentation pattern of [M + HCOO]^-^ adducts in the negative ion mode. The most abundant fragment ion observed in these analyses consisted of the [M – CH_3_ – HCOO]^-^ ion obtained through the loss of a methyl group from the phosphocholine headgroup, along with the formate adduct [10], along with an ion at *m/z* 168, which corresponded to the demethylated phosphocholine headgroup ([P-Cho – CH_3_ – H]^-^). The identity of the FA substituent could be detected by the presence of deprotonated FA anions in the negative ion mode. The structural information acquired in the positive and in the negative ion modes were combined so that the detailed structures of SM species could be reported. As was the case for Cer, when the appropriate amount of structural information could not be acquired, the condensed SM structure was presented.

A total of 5 IPC species were identified in the negative ion mode, forming mostly [M-H]^-^ adducts. Even though IPC and PI share the same headgroup (*myo*-inositol), these two lipid classes present different fragmentation patterns. The MS/MS analysis of both classes yields the fragment ion at *m/z* 241, corresponding to the *myo*-inositol cyclic phosphate, however, PI also generates abundant FA anions, while the fragmentation of IPC leads to the formation of an abundant ion at *m/z* 259, corresponding to *myo*-inositol phosphate anion. Another two abundant peaks observed through the MS/MS analysis of IPC, but not PI, correspond to the neutral loss of the *myo-*inositol headgroup ([M – 180 – H]^-^) and of the neutral loss of a dehydrated *myo*-inositol headgroup ([M – 162 – H]^-^) (Figure S8). Unfortunately, this analysis was insufficient to provide the detailed structure of the IPC species found in this study. All IPC species were reported in the condensed format. By far the most abundant IPC species found in *T. cruzi* amastigotes was IPC (34:1) at *m/z* 778.5 (Figure S8). This species has also been reported as the most abundant IPC in *Leishmania spp*. and in *T. brucei*, suggesting a remarkably conserved IPC synthesis pathway between these organisms [11].

**Triglyceride and diglyceride composition:** A total of 328 TG species were identified in the positive ion mode forming either [M+H]^+^, [M+Na]^+^, or [M+NH_4_]^+^ ions in a calculated mass range of 414.2 – 1018.9 a.m.u. The MS/MS analysis of TG molecular species forming [M+NH_4_]^+^ yielded abundant structural information pertaining to the identity of the FA substituents present in each of these species. The most abundant peaks observed through the fragmentation of ammoniated TG species in the positive ion mode corresponded to fragment ions derived from the neutral loss of individual FA substituents from the parent ion, as well as abundant peaks corresponding to dehydroxylated FA cations. The identity of the FA substituents could be further validated through the detection of dehydroxylated monoacylglyceride fragment ions, generated from the loss of two FA moieties, a hydroxyl group, along with the ammonium adduct from the parent ion. Taken together, the analysis of [M+NH_4_]^+^ TG ions yielded enough structural information for the for the FA identity of individual TG molecular species to be determined. The MS/MS analysis of TG molecular species forming [M + H]^+^ wasn’t as informative as [M+NH_4_]^+^ TGs, yielding mostly only fragment ions corresponding to the neutral loss of individual FA substituents. As was the case for glycerophospholipids, we did not conduct any experiments to verify the regiospecific location of the FA moieties in our TG analyses.

**Supporting References**

1. Richmond GS, Gibellini F, Young SA, Major L, Denton H, Lilley A, et al. Lipidomic analysis of bloodstream and procyclic form Trypanosoma brucei. Parasitology. 2010;137(9):1357-92. doi: 10.1017/S0031182010000715. PubMed PMID: 20602846; PubMed Central PMCID: PMCPMC3744936.

2. Serricchio M, Schmid AW, Steinmann ME, Sigel E, Rauch M, Julkowska D, et al. Flagellar membranes are rich in raft-forming phospholipids. Biol Open. 2015;4(9):1143-53. doi: 10.1242/bio.011957. PubMed PMID: 26276100; PubMed Central PMCID: PMCPMC4582118.

3. Smith TK, Butikofer P. Lipid metabolism in Trypanosoma brucei. Mol Biochem Parasitol. 2010;172(2):66-79. doi: 10.1016/j.molbiopara.2010.04.001. PubMed PMID: 20382188; PubMed Central PMCID: PMCPMC3744938.

4. Zhang K, Beverley SM. Phospholipid and sphingolipid metabolism in Leishmania. Mol Biochem Parasitol. 2010;170(2):55-64. doi: 10.1016/j.molbiopara.2009.12.004. PubMed PMID: 20026359; PubMed Central PMCID: PMCPMC2815228.

5. Ferguson MA, Brimacombe JS, Brown JR, Crossman A, Dix A, Field RA, et al. The GPI biosynthetic pathway as a therapeutic target for African sleeping sickness. Biochim Biophys Acta. 1999;1455(2-3):327-40. PubMed PMID: 10571022.

6. Villas Boas MH, Lara LS, Wait R, Bergter EB. Identification of plasmenylethanolamine as a major component of the phospholipids of strain DM 28c of Trypanosoma cruzi. Mol Biochem Parasitol. 1999;98(2):175-86. PubMed PMID: 10080387.

7. Weingartner A, Kemmer G, Muller FD, Zampieri RA, Gonzaga dos Santos M, Schiller J, et al. Leishmania promastigotes lack phosphatidylserine but bind annexin V upon permeabilization or miltefosine treatment. PLoS One. 2012;7(8):e42070. doi: 10.1371/journal.pone.0042070. PubMed PMID: 22870283; PubMed Central PMCID: PMCPMC3411662.

8. Ramakrishnan S, Serricchio M, Striepen B, Butikofer P. Lipid synthesis in protozoan parasites: a comparison between kinetoplastids and apicomplexans. Prog Lipid Res. 2013;52(4):488-512. doi: 10.1016/j.plipres.2013.06.003. PubMed PMID: 23827884; PubMed Central PMCID: PMCPMC3830643.

9. Sutterwala SS, Hsu FF, Sevova ES, Schwartz KJ, Zhang K, Key P, et al. Developmentally regulated sphingolipid synthesis in African trypanosomes. Mol Microbiol. 2008;70(2):281-96. doi: 10.1111/j.1365-2958.2008.06393.x. PubMed PMID: 18699867; PubMed Central PMCID: PMCPMC2629665.

10. Kerwin JL, Tuininga AR, Ericsson LH. Identification of molecular species of glycerophospholipids and sphingomyelin using electrospray mass spectrometry. J Lipid Res. 1994;35(6):1102-14. PubMed PMID: 8077849.

11. Hsu FF, Turk J, Zhang K, Beverley SM. Characterization of inositol phosphorylceramides from Leishmania major by tandem mass spectrometry with electrospray ionization. J Am Soc Mass Spectrom. 2007;18(9):1591-604. doi: 10.1016/j.jasms.2007.05.017. PubMed PMID: 17627842; PubMed Central PMCID: PMCPMC2065762.
