# Supplementary figures and images for: Host triacylglycerols shape the lipidome of intracellular trypanosomes and modulate their growth
Source: PLoS Pathog. 2017 Dec 27;13(12):e1006800. doi: 10.1371/journal.ppat.1006800 (PMC5760102; doi:10.1371/journal.ppat.1006800)

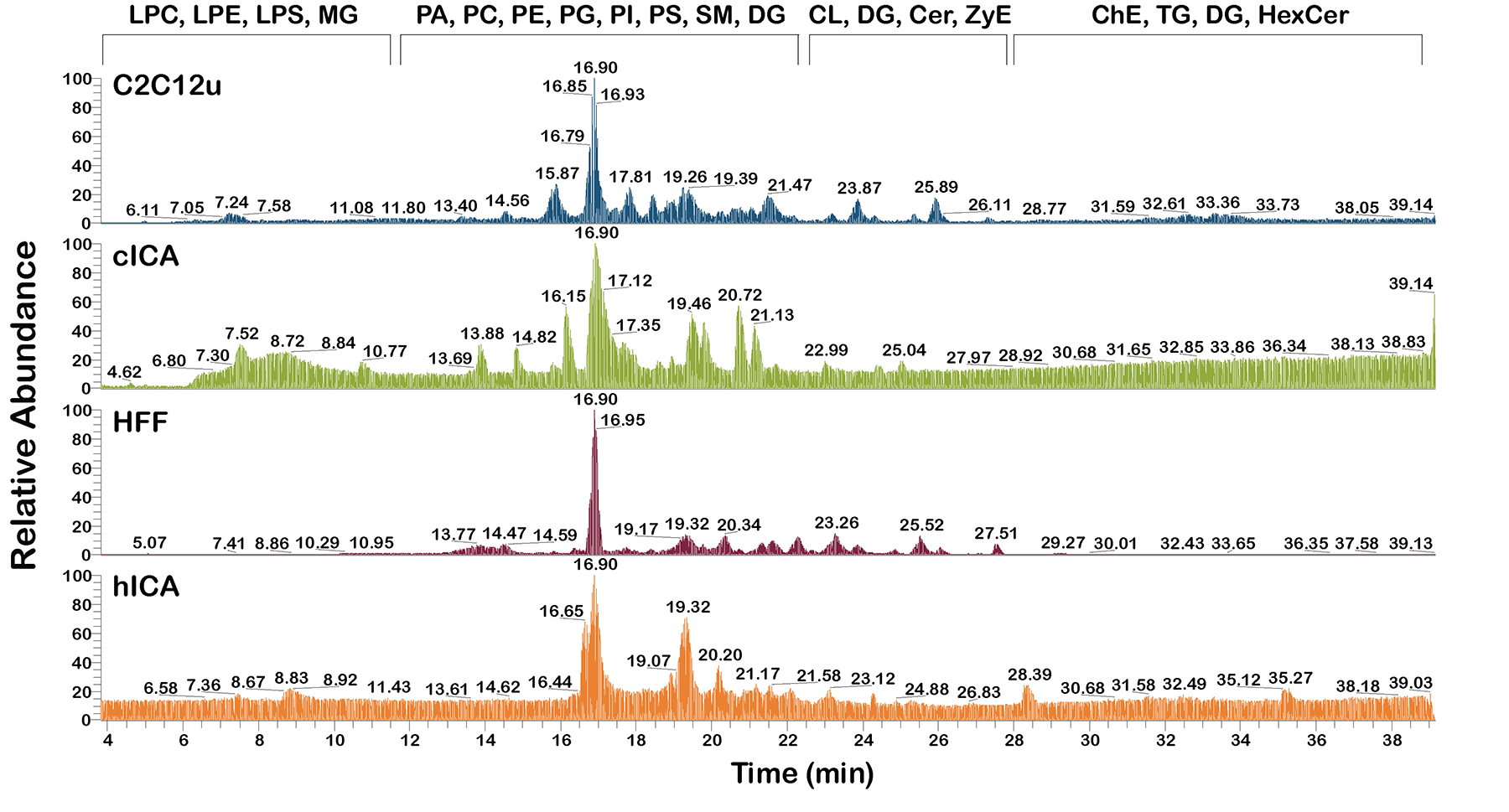

Supplement: S1 Fig — Negative ion mode base peak chromatogram of C2C12, HFF, and cognate T. cruzi amastigote (cICA and hICA, respectively). The major lipid subclasses eluting at different retention times (min) are indicated above the chromatogram. (TG–triacylglycerol, DG–diacylglycerol, Cer–ceramide, CerG–hexosylceramide, SM–sphingomyelin, LPC–lysophosphatidylcholine, PC–phosphatidylcholine, LPE–lysophosphatidylethanolamine, PE–phosphatidylethanolamine, LPS–lysophosphatidylserine, PS–phosphatidylserine, PI–phosphatidylinositol, PG–phosphatidylglycerol). (TIF) [file ppat.1006800.s002.tif]

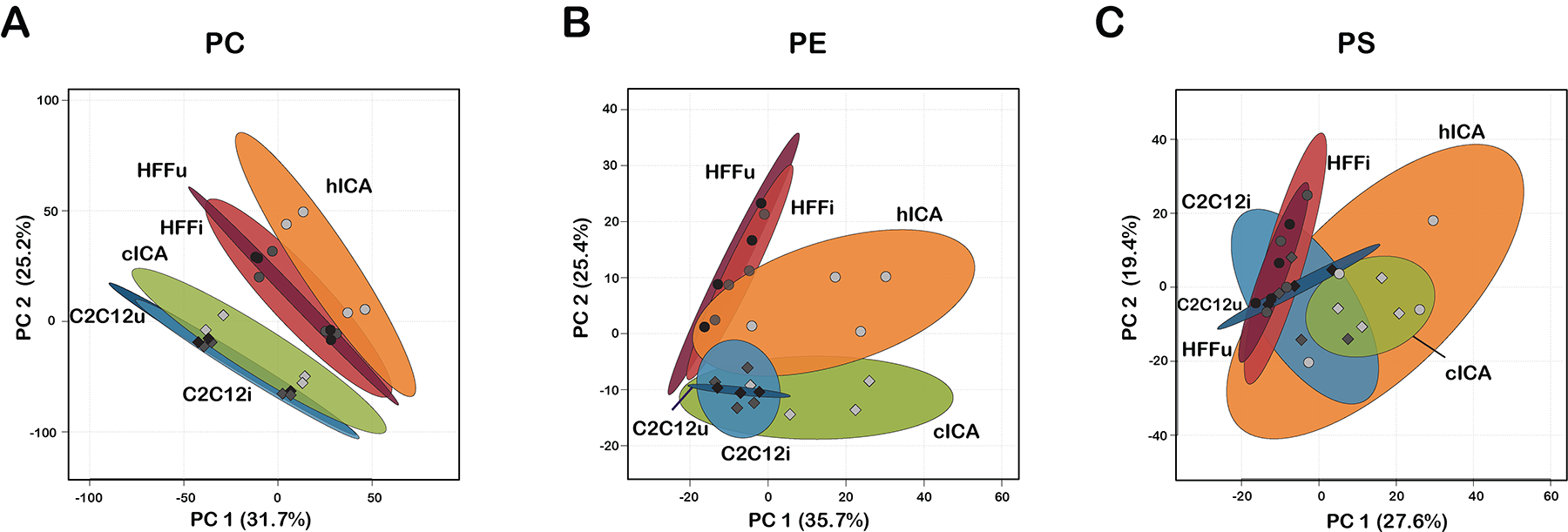

Supplement: S2 Fig — Principle component analysis of lipid species are plotted for (A) PC, (B) PE, and (C) PS subclasses. The first two principle components are plotted (PC1 and PC2) with proportion of variance for each component shown in parenthesis. Each sample is represented and the 95% confidence interval indicated in shaded circle. (TIF) [file ppat.1006800.s003.tif]

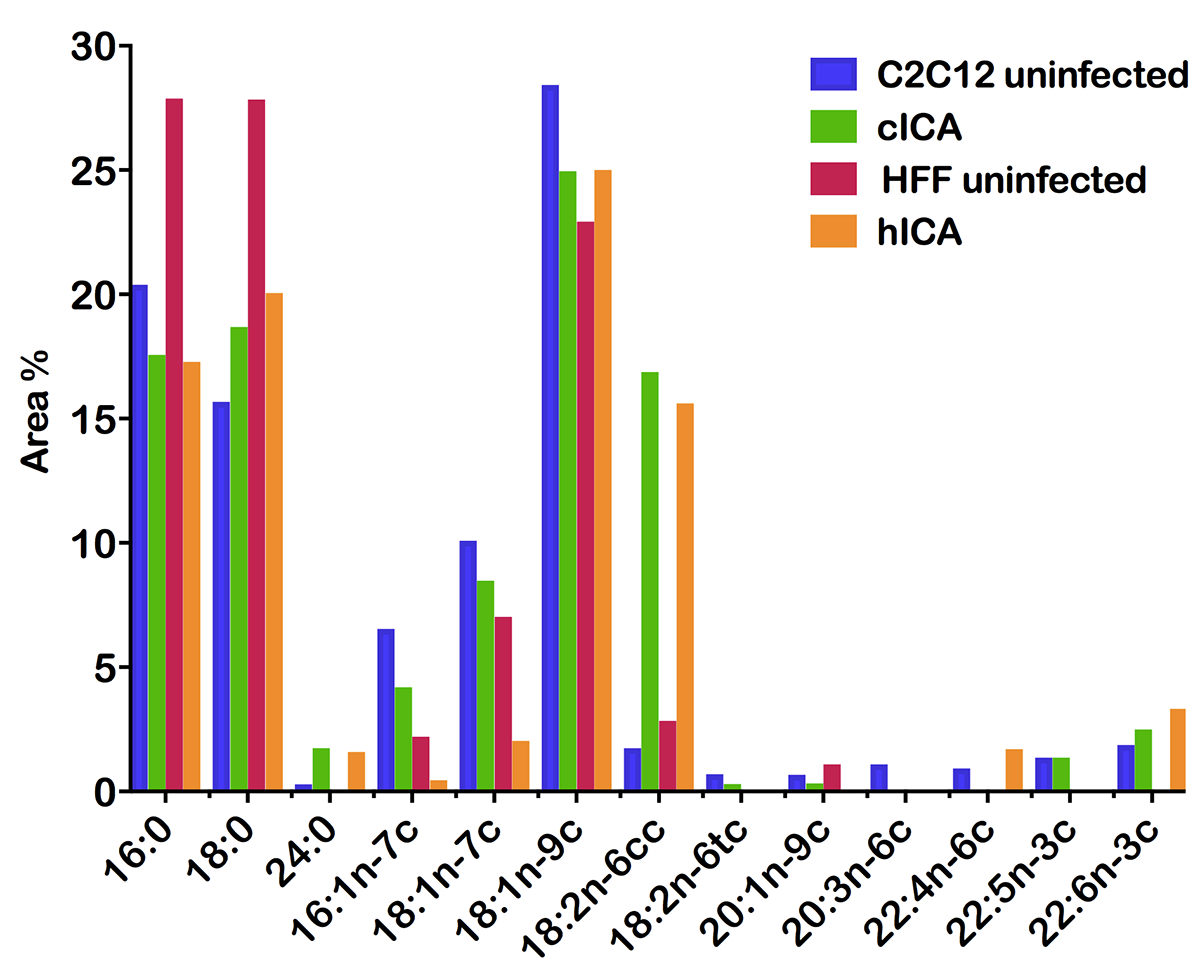

Supplement: S3 Fig — Esterified FA from lipid extracts were hydrolyzed with ammonium hydroxide at 37°C for 1 h, and methylated with 0.5 N methanolic-HCl for 1 h at 85°C. Methylated FA were recovered by dichloromethane:water partitioning and analyzed by GC-FID. Area % is plotted for each FA moiety detected. Data are from a representative experiment. (TIF) [file ppat.1006800.s004.tif]

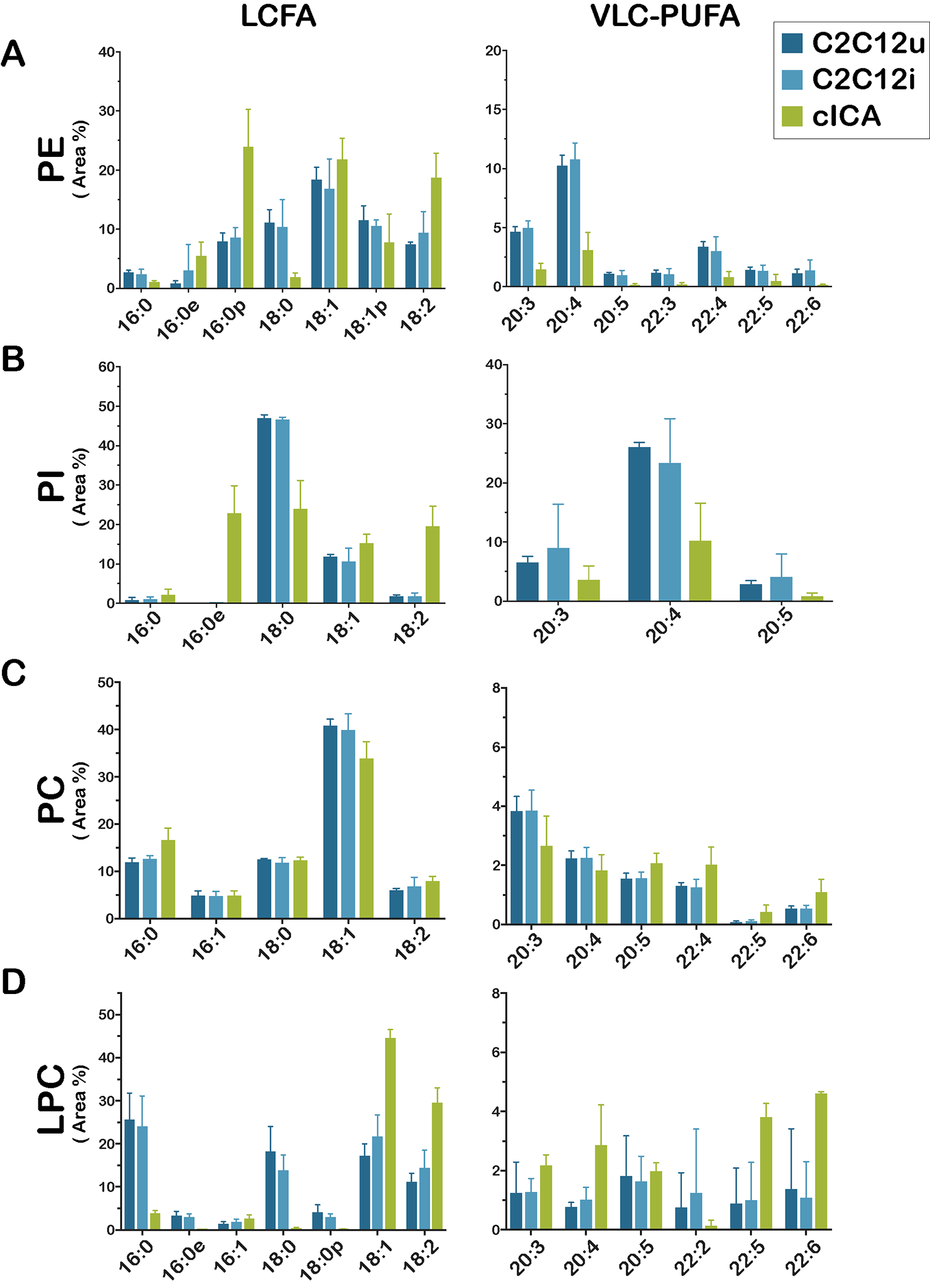

Supplement: S4 Fig — FA area % is plotted for long-chain fatty acid (LCFA) and very long-chain polyunsaturated fatty acid (VLC-PUFA) of (A) PC, (B) LPC, (C) PE and (D) PI for samples derived from C2C12 host cells (HFF main text, Fig 4). Data are represented as mean ± standard deviation. (TIF) [file ppat.1006800.s005.tif]

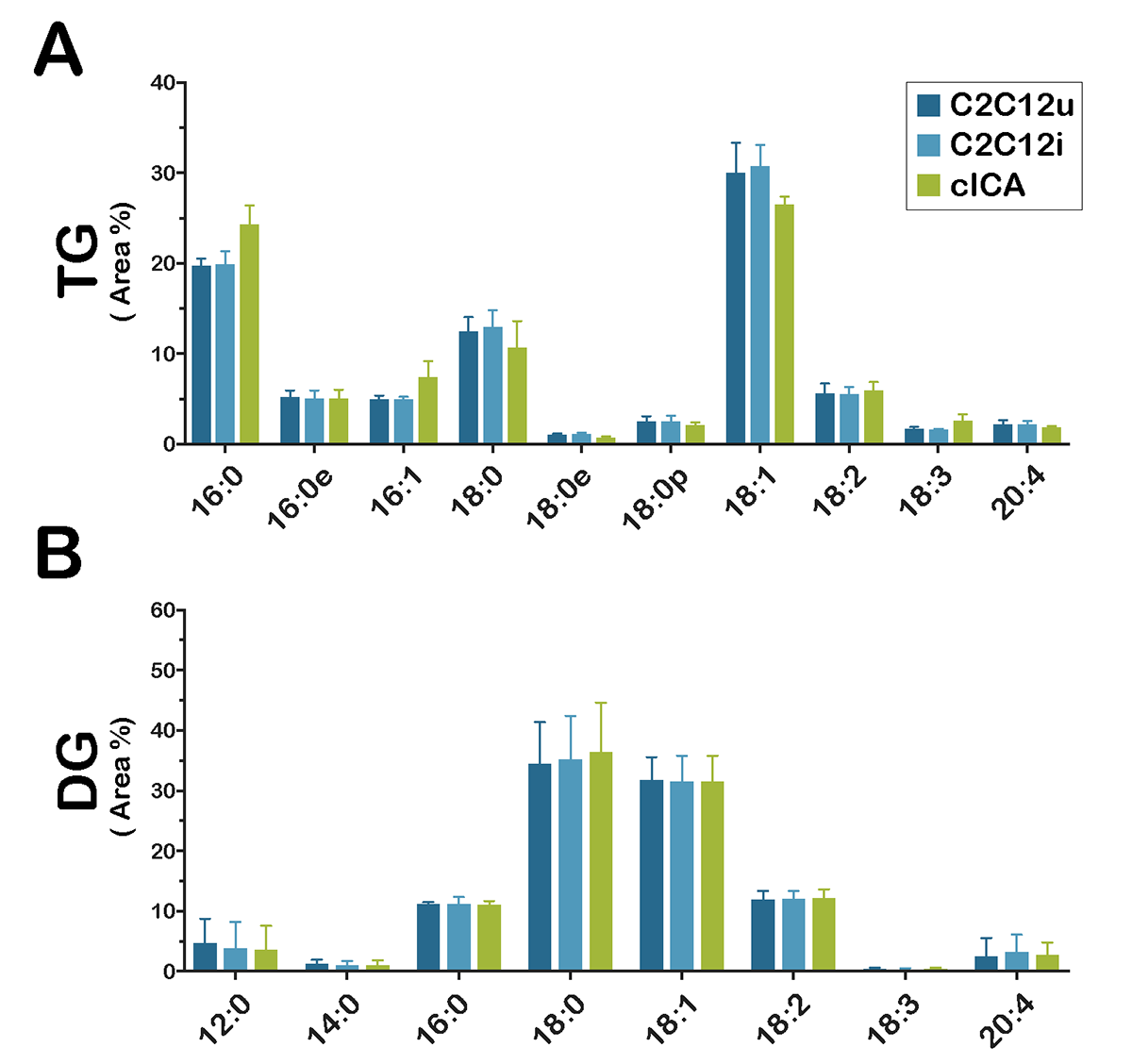

Supplement: S5 Fig — FA area % is plotted for (A) TG and (B) DG classes for samples derived from C2C12 host cells; (HFF main text plotted in Fig 5). Data are represented as mean ± standard deviation. (TIF) [file ppat.1006800.s006.tif]

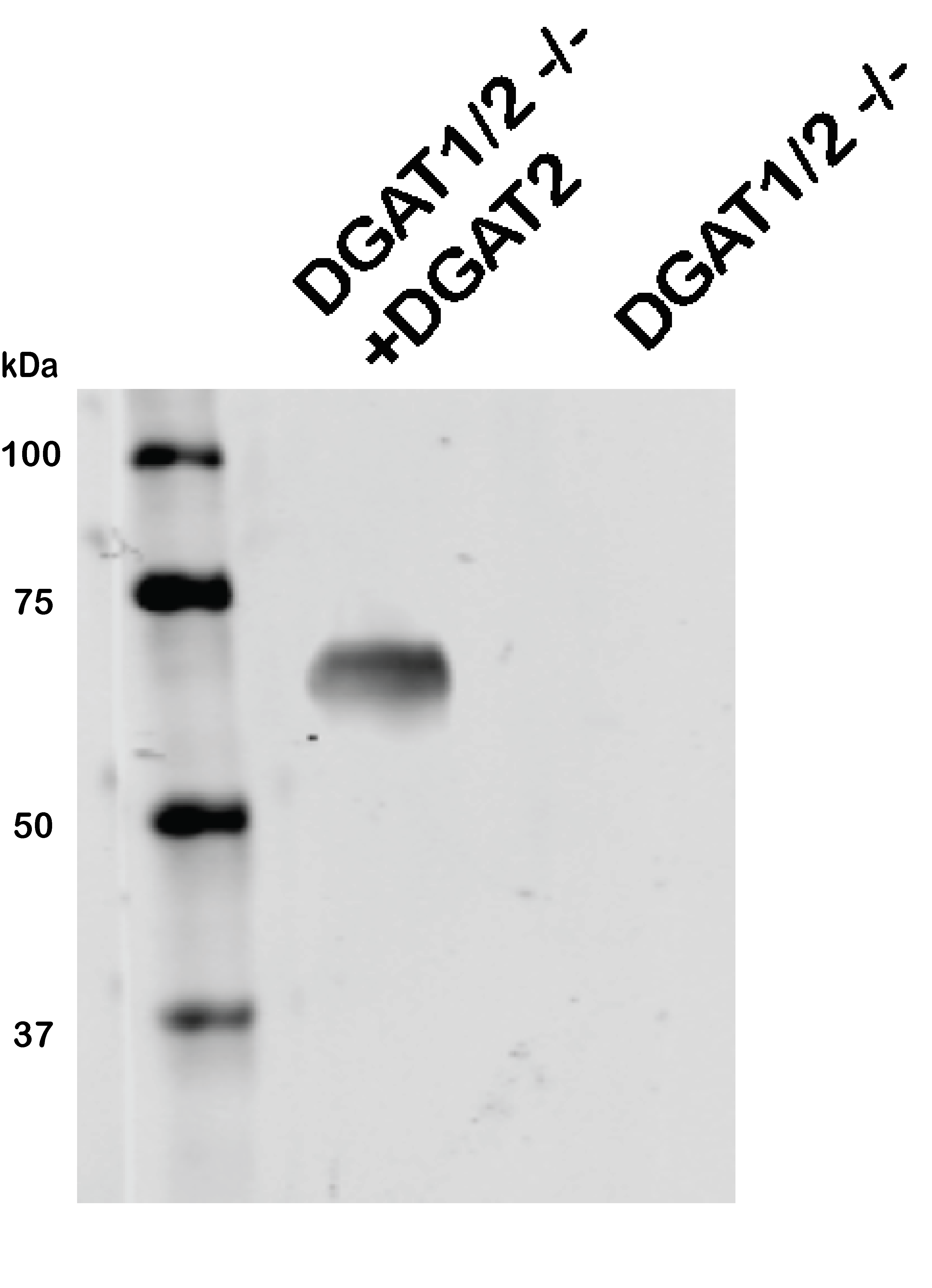

Supplement: S6 Fig — Protein expression of Myc-DDK-DGAT2 was confirmed by immunoblotting using the FLAG M2 antibody. (TIF) [file ppat.1006800.s007.tif]

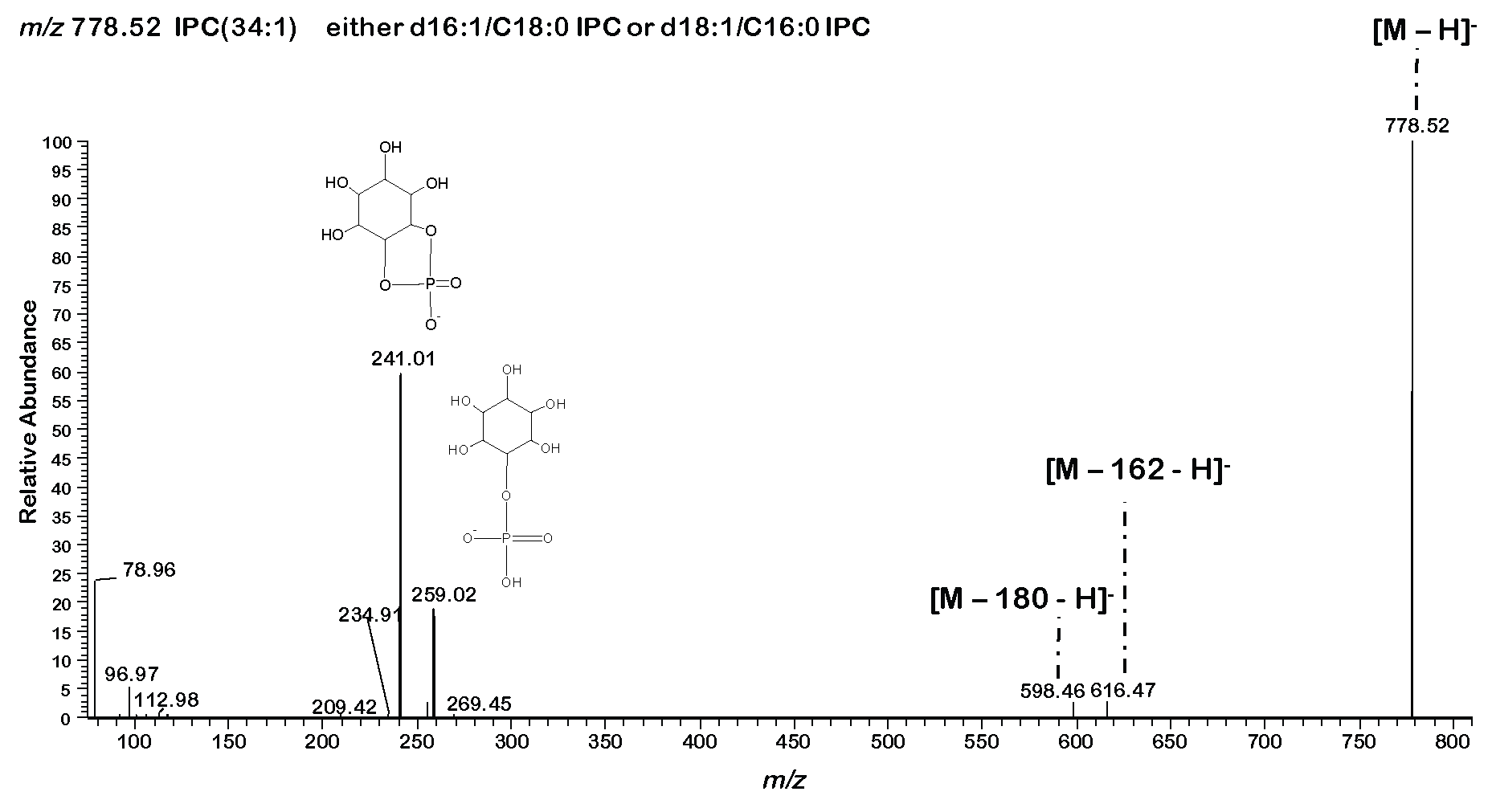

Supplement: S7 Fig — Representative MS/MS spectrum of the most abundant IPC species identified in T. cruzi was acquired from lipid extracts of TCT as described in the supplementary methods section of the manuscript (Supporting Information: Supplemental Methods). Fragment ions from MS/MS analysis are indicated in the figure, according to previously published data [74]. (TIF) [file ppat.1006800.s008.tif]
